# Transcription Elongation Factor GreA Plays a Key Role in Cellular Invasion and Virulence of *Francisella tularensis* subsp. *novicida*

**DOI:** 10.1038/s41598-018-25271-5

**Published:** 2018-05-02

**Authors:** Guolin Cui, Jun Wang, Xinyi Qi, Jingliang Su

**Affiliations:** Key Laboratory of Animal Epidemiology and Zoonosis, Ministry of Agriculture, College of Veterinary Medicine, China Agricultural University, Beijing, 100193 China

## Abstract

*Francisella tularensis* is a facultative intracellular Gram-negative bacterium that causes the zoonotic disease tularemia. We identified the transcription elongation factor GreA as a virulence factor in our previous study, but its role was not defined. Here, we investigate the effects of the inactivation of the *greA* gene, generating a *greA* mutant of *F. tularensis* subsp. *novicida*. Inactivation of *greA* impaired the bacterial invasion into and growth within host cells, and subsequently virulence in mouse infection model. A transcriptomic analysis (RNA-Seq) showed that the loss of GreA caused the differential expression of 196 bacterial genes, 77 of which were identified as virulence factors in previous studies. To confirm that GreA regulates the expression of virulence factors involved in cell invasion by *Francisella*, FTN_1186 (*pepO*) and FTN_1551 (*ampD*) gene mutants were generated. The *ampD* deletion mutant showed reduced invasiveness into host cells. These results strongly suggest that GreA plays an important role in the pathogenesis of *Francisella* by affecting the expression of virulence genes and provide new insights into the complex regulation of *Francisella* infection.

## Introduction

*Francisella tularensis* is a Gram-negative bacterium that causes zoonotic tularemia in humans and a large number of animal species^1^. Humans can be infected by multiple routes, including the inhalation of bacteria-containing droplets or dust, the consumption of contaminated food or water, and arthropod bites^1,2^. Four subspecies, designated *F. tularensis* subsp. *tularensis* (type A), subsp. *holarctica*, subsp. *mediasiatica*, and subsp. *novicida*, have been identified, with the different genetic backgrounds, virulence, and geographic distributions^3–5^. Subspecies *tularensis* is a highly virulent pathogen that can cause lethal infections in humans or animals after the inhalation of only several to tens of bacteria^1^. Based on its extreme infectivity, it is classified as a Category A bioweapon agent, with a risk of misuse. Because of this classification, biosafety level 3 (BSL3) facilities are required to study the type A strain. Subspecies *novicida* is not normally pathogenic to humans, but can infect mice, causing a tularemia-like disease. Interestingly, it shares ≥97.7% sequence identity and homologous virulence factors with subsp. *tularensis*^6^. Therefore, subsp. *novicida* has been extensively used as a model in which to study the virulent subsp. *tularensis*, with the advantage of its easy genetic manipulation without the requirement for BSL3.

Because it is a facultative intracellular bacterium, *F. tularensis* infection involves both extra- and intracellular processes. The bacterium invades and replicates within the host cell, resulting in its systemic dissemination. Its adaptation to changing environments requires the timely activation and/or repression of the expression of many virulence genes, and gene transcription changes have been detected in *F. tularensis* during intracellular infection^1,3,7–10^. Several virulence regulators of *F. tularensis*, including MglA, SspA, FevR (PigR), PmrA (QseB), Hfq, and MigR, have been shown to contribute to gene transcriptional regulation. MglA and SspA form a complex and positively control the expression of virulence genes, including all the *Francisella* pathogenicity island (FPI) genes^11,12^. FevR is activated by contact with the MglA/SspA complex and positively regulates genes that contain a 7-bp sequence element in their promoters^13^. Unlike those three regulators, PmrA and Hfq function individually. PmrA, an orphan response regulator, regulates FPI genes, *fevR*, and *migR*^14^, and Hfq negatively regulates several virulence genes^15^. However, MigR indirectly regulates the *igl* operon by downregulating the expression of FevR^16^. Interestingly, the regulation of gene transcription in *F. tularensis* appears to be unique. There is no complete two-component regulatory system (TCS) in subsp. *tularensis* or subsp. *holarctica*, whereas two complete TCSs are present in subsp. *novicida*^17^. Furthermore, only one alternative sigma factor (σ^32^) for the *F. tularensis* stress response has been found so far^18^.

GreA is an evolutionarily conserved transcription factor distributed widely in prokaryotes. It has a long coiled-coil domain that binds in the secondary channel of the RNA polymerases (RNAPs). Active GreA suppresses transcriptional pauses by rescuing the backtracked transcription-elongation complexes, stimulating RNAP promoter escape and enhancing transcriptional fidelity^19–23^. In pathogenic *Streptococcus pneumoniae*, GreA is a rate-limiting factor in the expression of highly expressed genes, and the disruption of *greA* alters gene expression and reduces the growth of the bacterium^24^. GreA is also recognized as a stress protein, induced in different stressful environment, including under heat shock, salt stress, and oxidative stress, in several bacterial species^25–31^.

The *greA* gene was first identified as a virulence-associated gene in *Yersinia pestis* using signature-tagged mutagenesis^32^. In a mouse model, a transposon insertion mutant at the *F. tularensis* LVS *greA* gene was significantly outcompeted by the wild-type strain in a mixed infection^33^. This prompted us to investigate the role of GreA in the infectivity in *Francisella*. In this study, we demonstrate that *greA* is critical for bacterial invasion into and growth within host cells, and the *in vivo* fitness of *F. novicida*. We also show that the loss of GreA significantly alters the transcription profiles of a variety of genes, including a subset of previously identified virulence genes. Last, we demonstrate that *ampD* is required for bacterial entry into the host cells.

## Results

### Genes in the *greA* cluster of *F. novicida* are cotranscribed

**Figure 1:**
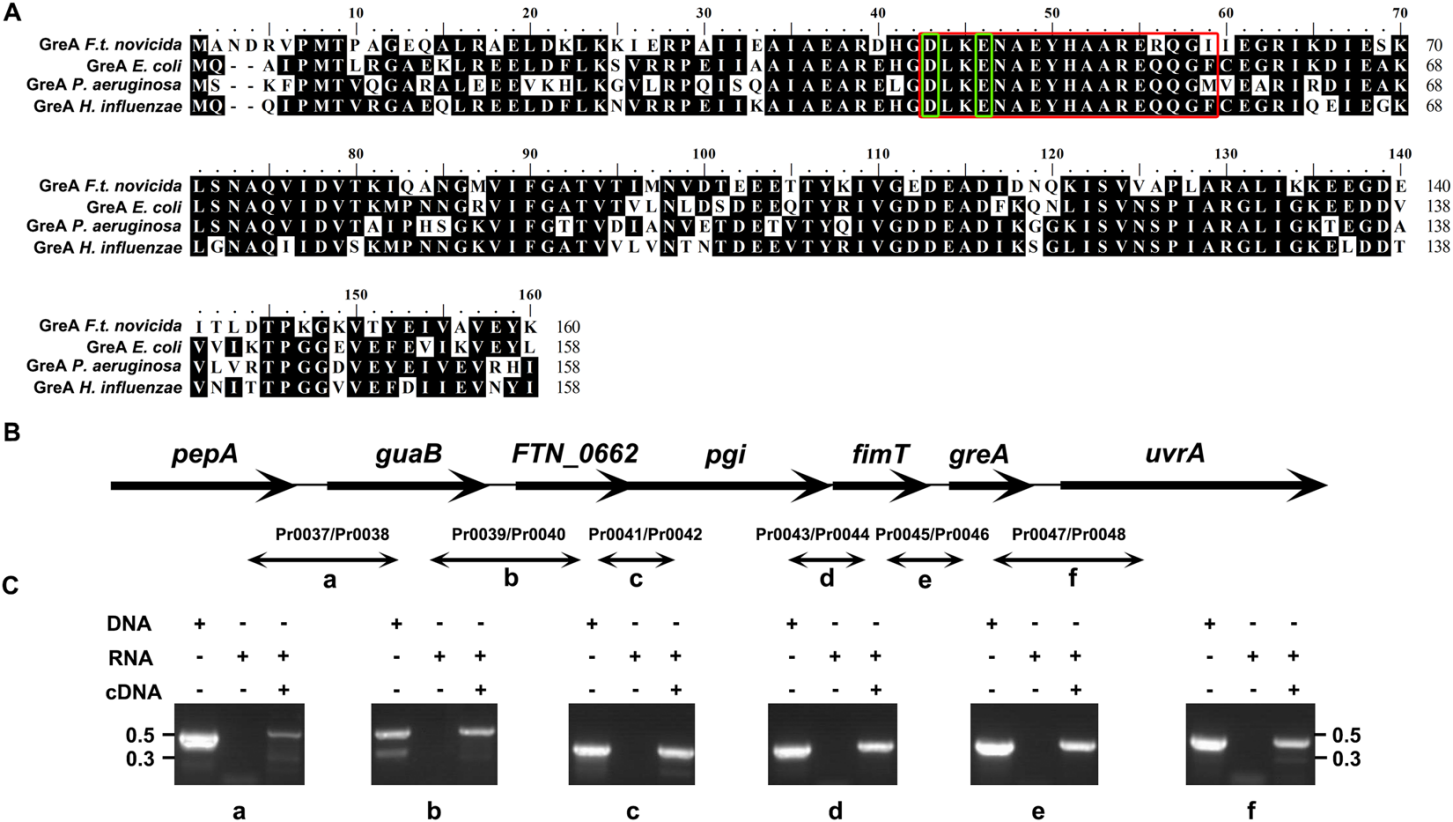
Analysis of the *F. novicida greA* locus. (**A**) Alignment of four Gram-negative bacterial GreA amino acid sequences using the MegAlign program of the DNAstar Lasergene package (version 10). Identical amino acid residues are shown in black. The cross-link with the RNA 3′-terminus is shown in a red box and the conserved acidic residues (D43 and E46) required for GreA activity are shown in green boxes. (**B**) Schematic illustration of the gene arrangement at the *greA* locus. (**C**) Cotranscription of the *greA* locus genes determined with RT–PCR. The *pepA-–guaB* (a), *guaB–*FTN_0662 (b), FTN_0662*–pgi* (c), *pgi–fimT* (d), *fimT–greA* (e), and *greA–uvrA* (f) junctions were amplified using DNA, DNA-free RNA, or cDNA as the template. The images were acquired by the gel imaging system (LIUYI, Beijing, China). The experiment was repeated twice. The sizes of the molecular markers are indicated at the side in kbp.

A genomic sequence analysis showed that *F. novicida* contains only one Gre factor (GreA) encoded by the FTN_0665 (*greA*) gene. It consists of 160 amino acids, with 100% identity to the corresponding proteins of the other three *F. tularensis* subspecies. Alignment of the amino acid sequences of these GreA proteins with an array of GreA sequences of other Gram-negative bacterial species revealed two conserved acidic residues (D43 and E46) that are required for its activity (Fig. 1A)^34^. As shown in Fig. 1B, the *greA* gene is located downstream from the *fimT* gene (FTN_0664, encoding type IV pili fiber building block protein) and upstream from the *uvrA* gene (FTN_0666, encoding a DNA excision repair enzyme, subunit A of the UvrABC system), and the gene organization is conserved among the *F. tularensis* subspecies. Genome annotation predicts that the five upstream genes (FTN_0660 to FTN_0664) and the downstream *uvrA* gene are transcribed in the same orientation, suggesting the potential cotranscription of these genes. We tested this possibility by amplifying the RNA transcripts that bridged the adjacent genes, using the indicated primer pairs (Fig. 1B and Table S1), and the intergenic fragments produced had the predicted sequences (Fig. 1C). These results indicate that, under *in vitro* culture conditions, *greA* is expressed primarily as part of an operon containing seven genes.

### Deletion of *greA* influences the *in vitro* physiology of *F. novicida*

**Figure 2:**
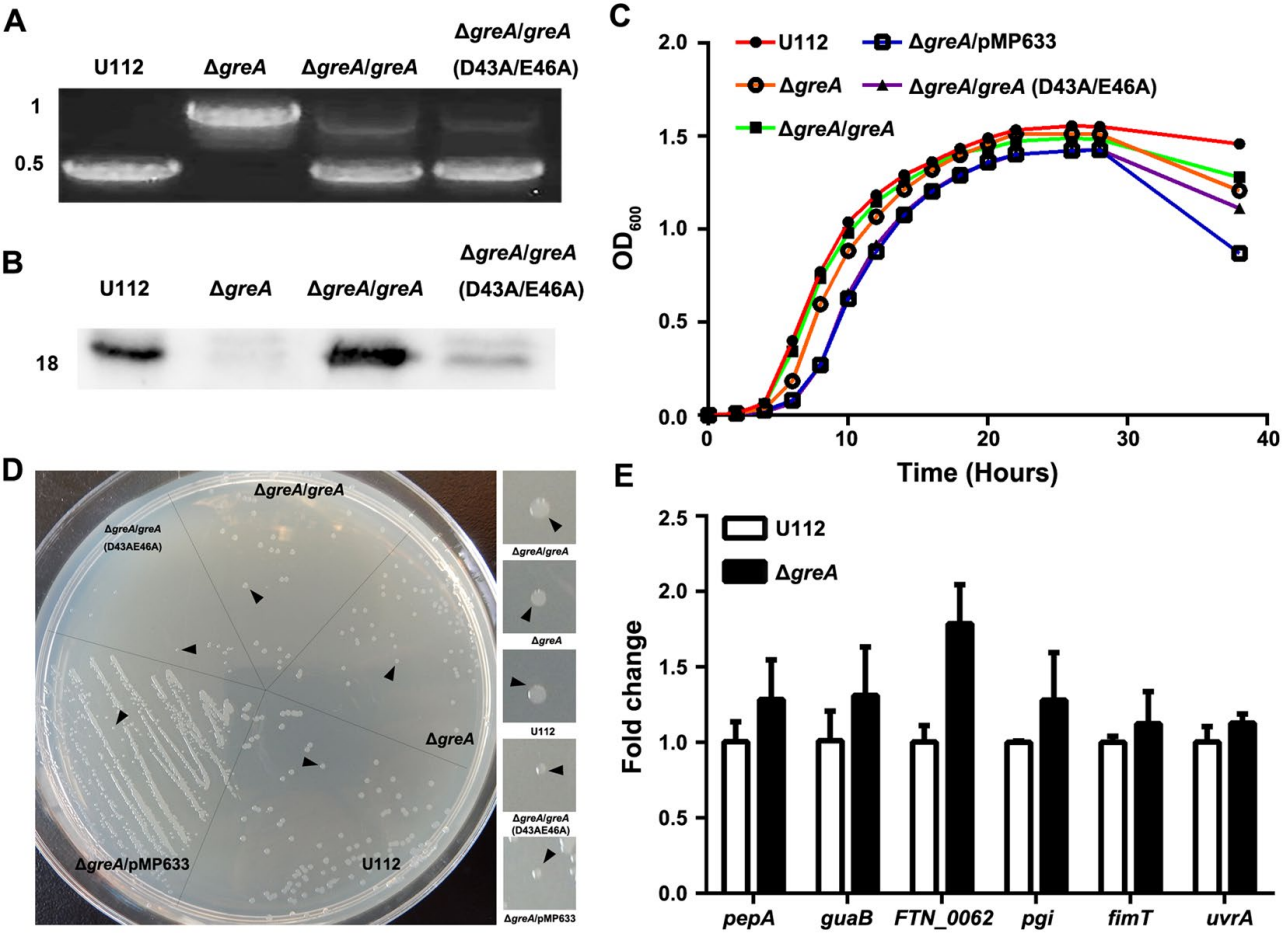
Contribution of GreA to the growth of *F. novicida*. (**A**) Deletion of *greA* gene in *F. novicida* strain U112 was confirmed with PCR using primer pair Pr0023/Pr0024. The images were acquired by the gel imaging system (LIUYI). Sizes of molecular markers are indicated at the left in kbp. (**B**) Loss of GreA protein in *F. novicida* was detected with western blotting using a mouse polyclonal antibody raised against recombinant GreA protein. The image was acquired by the automatic chemiluminescence image analysis system (Tanon). Size of the protein is indicated at the left in kDa. (**C**) Growth kinetics of wild-type strain U112 and its derivatives in TSB. The values represent the means ± SD (n = 3) from one of the three independent experiments. (**D**) Growth of wild-type strain U112 and its derivatives on TSA plates. The image was acquired with the digital camera (Canon, Janpan). (**E**) Transcription of *greA* locus genes in wild-type strain U112 and the Δ*greA* mutant. Data are the relative transcription levels of each target gene normalized to that of the 16S rRNA gene. Results are shown as the mean fold changes relative to the wild-type U112 strain ± SD (n = 3) from one of the three independent experiments. Statistical significance was determined with unpaired Student’s *t* test.

Because GreA is known to play important roles in the growth and physiology of several bacteria, including *Escherichia coli* and *S. pneumonia*^24,35^, we wondered if this was also the case in *Francisella*. We constructed a Δ*greA* mutant of *F. novicida* U112 by replacing the *greA* gene with a kanamycin resistance (kan^r^) cassette, and the disruption of *greA* in the mutant was verified with PCR amplification (Fig. 2A). An *in trans*-complemented strain Δ*greA/greA* was also generated and the restoration of GreA expression was confirmed with western blotting (Fig. 2B). Under *in vitro* culture conditions, Δ*greA* displayed a slower growth rate and reached a lower final optical density in tryptic soy broth (TSB) culture than the wild type (Fig. 2C). The doubling time of the Δ*greA* mutant increased significantly during the first 10 h after inoculation into TSB (56 ± 1.7 min for Δ*greA* mutant versus 42 ± 2 min for wild-type, *p* < 0.001). Consistent with this, the Δ*greA* mutant produced smaller colonies on tryptone soy agar (TSA) plates 24 h after inoculation (Fig. 2D). *In trans* complementation with intact *greA* restored the growth of the Δ*greA* mutant in both liquid medium and on agar plates (Fig. 2C,D). To confirm that the catalytic activity of GreA is required for bacterial growth, a D43A/E46A *greA* mutant was generated by substituting the two active amino acids with alanine (Fig. 2A,B). Consistent with the importance of these two acidic residues in GreA^34^, *in trans* complementation with the mutated *greA* gene did not restore normal growth to the Δ*greA* mutant (Fig. 2C,D). The transcripts of the adjacent genes (*pepA, guaB*, FTN_0662, *pgi*, *fimT*, *urvA*) in the operon were also analyzed with quantitative real-time reverse transcription PCR (qRT-PCR) and no significant changes (<2-fold) in the transcription levels of the six genes were observed (Fig. 2E), excluding the possible polar effect of the kan^r^ cassette. These data demonstrate the specific role of GreA in the *in vitro* growth of *F. novicida*.

**Figure 3:**
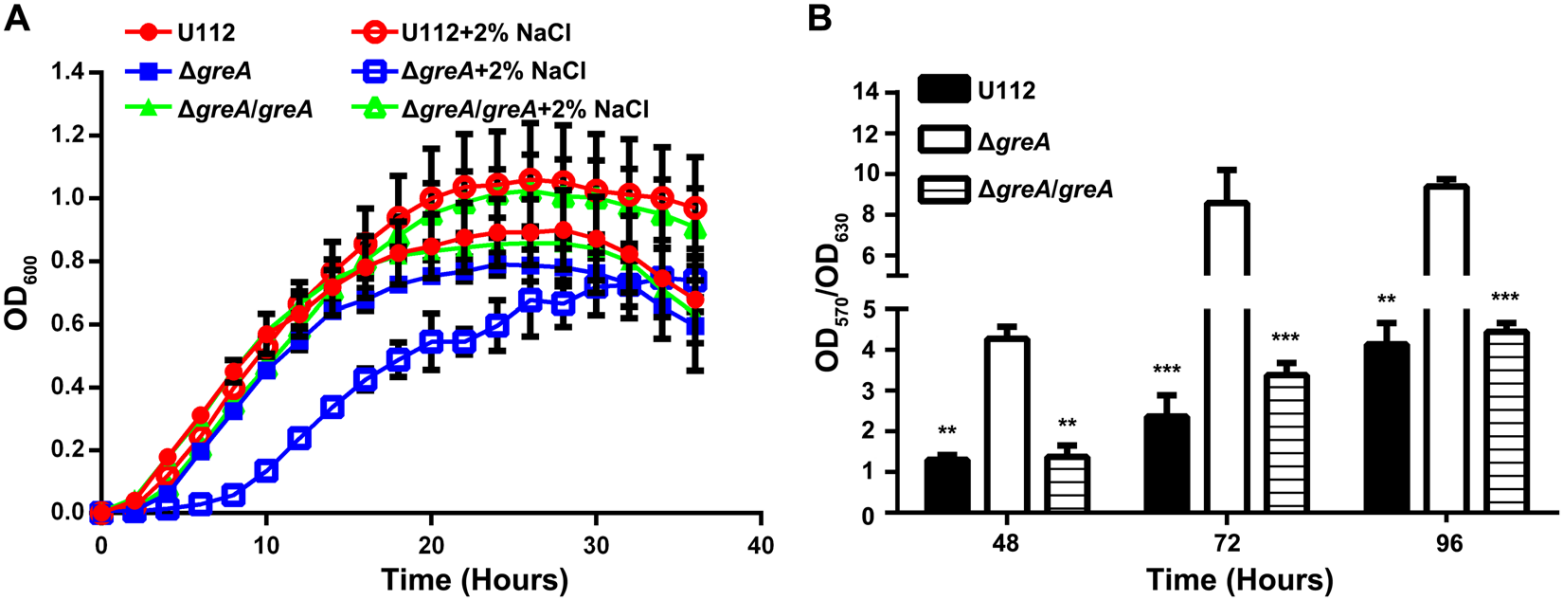
Bacterial stress tolerance and biofilm formation. (**A**) Growth kinetics of wild-type strain U112 and its derivatives in TSB with or without 2% NaCl. The values represent the means ± SD (n = 3) from one of the three independent experiments. (**B**) Biofilm formation by wild-type strain U112 and its derivatives in TSB in 96-well plates. Results are shown as OD_570_/OD_600_. The values were the means ± SD of the results from triplicate samples as compared with those of the Δ*greA* mutant. The experiment was repeated twice. Statistical significance was determined by one-way analysis of variance (ANOVA) test. **p < 0.01 and ***p < 0.001.

GreA is important in the resistance of *E. coli* to various stresses^25,26,30,31^. Therefore, we tested whether the deletion of *greA* affected the stress resistance of the mutant to high-temperature, oxidative, or osmotic stress. We observed no significant (*p* < 0.05) growth defect in the mutant during culture at 40 °C and no loss of resistance to H_2_O_2_. We noted that that addition of 2% NaCl enhanced the growth of the wild-type U112 strain, with a slightly higher optical density at a wavelength of 600 nm (OD_600_) than when grown in medium without NaCl supplementation (Fig. 3A). In contrast, the Δ*greA* mutant displayed slower growth in NaCl-supplemented medium, with a prolonged lag phase, and this growth defect was alleviated by *in trans* complementation (Fig. 3A). To examine whether the observed growth defect was attributable to the osmotic effect of NaCl or an ionic effect, we assessed the growth of wild-type U112 and its isogenic mutants in medium supplemented with a high concentration of sorbitol. All the strains showed similar growth defects in medium containing 12.5% sorbitol, which produces similar osmotic stress to 2% NaCl (Fig. S1), suggesting that the deletion of *greA* affects the maintenance of ion homeostasis in *F. novicida*.

Because biofilm formation is an important defense mechanism of bacteria under stress conditions and *F. novicida* is the only subspecies that has been shown to form a significant biofilm^36–38^, the ability of the Δ*greA* mutant to form a biofilm was examined. Intriguingly, after prolonged incubation, the Δ*greA* mutant formed a stronger biofilm than the wild-type U112 strain (Fig. 3B).

### Virulence of the *greA* mutant is attenuated in a mouse model

**Figure 4:**
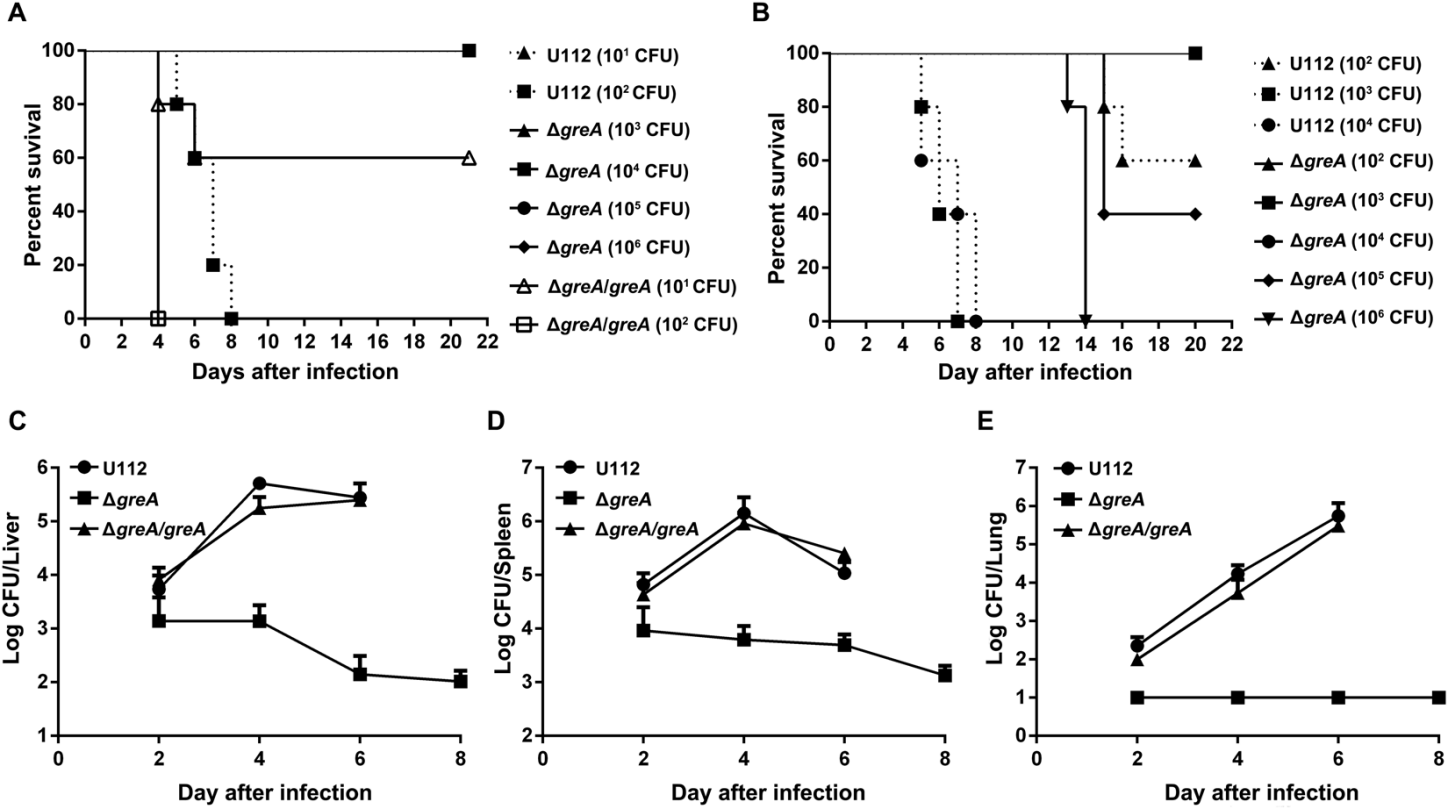
Impact of *greA* deletion on the *in vivo* growth and pathogenicity of *F. novicida*. (**A,B**) Survival of mice infected intranasally (n = 5) (**A**) or subcutaneously (n = 5) (**B**) with different doses of wild-type strain U112 or its derivatives. (**C–E**) Growth of wild-type strain U112 and its derivatives in the livers (**C**), spleens (**D**), and lungs (**E**) of mice after subcutaneous infection with doses of 10^3^ CFU/mouse of the wild-type strain U112 or Δ*greA*/*greA*, or 3 × 10^5^ CFU/mouse of the Δ*greA* mutant. All values are mean log CFU/organ ± SD (n = 5). *p* < 0.05 compared with the Δ*greA* group, based on log-transformed data at all time points. The experiment was repeated once. Statistical significance was determined by one-way analysis of variance (ANOVA) test.

To determine whether GreA is important for the *in vivo* pathogenicity of *F. novicida*, we assessed the virulence of the Δ*greA* mutant in mice. Groups of mice were infected with different numbers of bacteria, and their survival was recorded for 21 days. Mice infected intranasally with 10^2^ colony-forming units (CFU) of wild-type strain U112 died during the observation period, whereas all the mice infected with the Δ*greA* mutant at doses of 10^3^–10^6^ CFU survived (Fig. 4A). The *greA* deletion mutant also displayed significantly attenuated virulence after subcutaneous infection. The median lethal dose (LD_50_) of Δ*greA* mutant was 10^4.83^ CFU when calculated with the Reed–Muench method^39^, in contrast to that of 10^2.17^ CFU for the wild-type U112 strain (Fig. 4B). These results demonstrate that the virulence of the Δ*greA* mutant is highly attenuated.

We next investigated the fate of the Δ*greA* mutant inside the host by infecting mice subcutaneously with 3 × 10^5^ CFU of the Δ*greA* mutant or 10^3^ CFU of the wild-type U112 strain or the *in trans*-complemented strain (Δ*greA/greA*), and quantitatively assessed the growth kinetics of these bacteria. As shown in Fig. 4C–E, the wild-type U112 strain replicated and dispersed efficiently in the internal organs of the mice, and the bacterial number increased from day 2 to day 6, reaching about 10^6^ CFU per organ. In contrast, the highest numbers of Δ*greA* mutant bacteria recovered from the mouse livers and spleens were significantly lower than those in the wild-type-U112-infected mice, although the infective dose was 300-fold higher than the wild-type strain U112 dose (Fig. 4C–E). Notably, the Δ*greA* mutant was not recovered from the lungs of the infected mice at all time points (limit of detection ≥ 10 CFU per lung), suggesting that its ability to cause systemic infection was also attenuated. *In trans* complementation of the *greA* gene significantly restored the *in vivo* virulence (Fig. 4A,B) and growth of the Δ*greA* mutant (Fig. 4C–E). Taken together, these results show that GreA contributes to the virulence of *F. novicida* in mice and its growth *in vivo*, confirming our previous finding that GreA is an important virulence determinant in *F. tularensis*^33^.

### Deletion of *F. novicida greA* gene impairs its ability to invade and replicate within host cells

**Figure 5:**
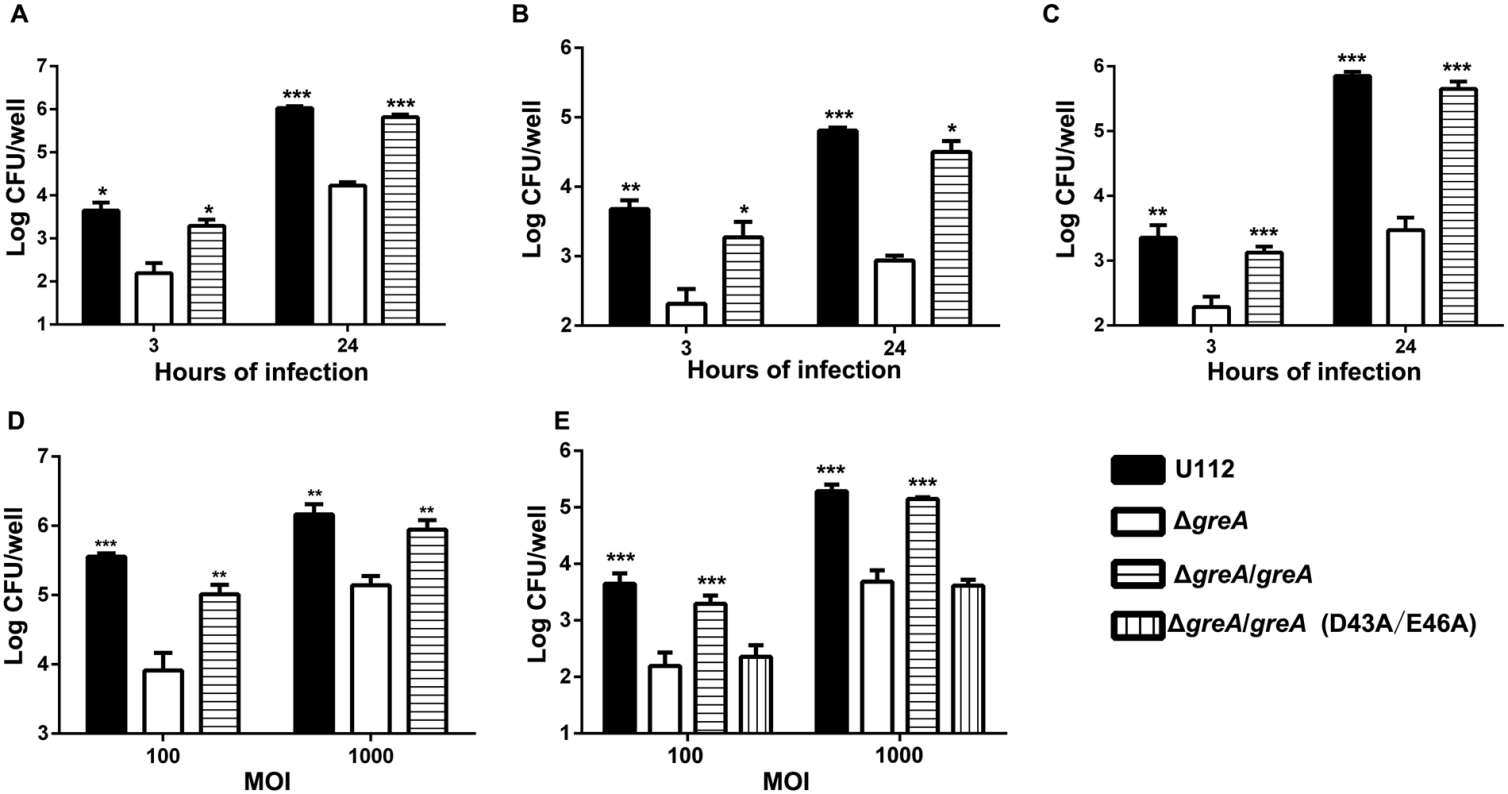
Entry and intracellular growth of wild-type strain U112 and its derivatives. (**A–C**) Intracellular growth of wild-type strain U112 and its derivatives. RAW264.7 (**A**), mouse bone-marrow-derived macrophages (**B**), and A549 (**C**) cell monolayers were infected with the wild-type U112 strain or its derivatives at an MOI of 100:1. At 3 and 24 h post infection, the cells were lysed and plated for enumeration. (**D**) Adhesion of the wild-type U112 strain and its derivatives to RAW264.7 cells. RAW264.7 cells were pretreated with 1 μg/ml cytochalasin D for 1 h before infection with the wild-type U112 strain or its derivatives at an MOI of 100:1 or 1000:1. After incubation for 2 h, the cells were lysed to enumerate the adherent bacteria CFU. (**E**) Invasion of RAW264.7 cells by the wild-type U112 strain or its derivatives. RAW264.7 cells were infected with the wild-type U112 strain or its derivatives at an MOI of 100:1 or 1000:1 for 2 h, and then treated with 100 μg/ml gentamycin for 1 h. The invasive bacteria were quantified by plating the cell lysates on TSA and counting the CFU. The values are mean log CFU/well ± SD of the results from triplicate samples as compared with those of the Δ*greA* mutant. The experiments were repeated at least twice. Statistical significance was determined by one-way analysis of variance (ANOVA) test. *p < 0.05, **p < 0.01, and ***p < 0.001.

To clarify the attenuation of the Δ*greA* mutant of *F. novicida* in mice, we assessed its infectivity using several cell models. We initially infected murine macrophage RWA264.7 cells and counted the intracellular bacteria by plating the lysates onto the agar plates. At 3 and 24 h after infection the intracellular bacterial numbers of the Δ*greA* mutant were significantly lower than those of the wild-type U112 strain (Fig. 5A). Meanwhile, the bacterial replication rate of the Δ*greA* mutant at 3–24 h post infection was lower as compared with the wild-type U112 strain (Fig. 5A). Similar patterns were observed in infected mouse bone-marrow-derived macrophage cultures (Fig. 5B) and human lung epithelial cell A549 cultures (Fig. 5C). These defects were restored in the complementation construct Δ*greA/greA* (Fig. 5A–C). To test the invasion defect, we performed cellular invasion experiments using RAW264.7 cells at different multiplicities of infection (MOIs). As shown in Fig. 5D, the cell-associated numbers of Δ*greA* mutant bacteria decreased 44-fold and 10-fold at MOIs of 100:1 and 1000:1, respectively, when the cells were pretreated with cytochalasin D to inhibit bacterial internalization, as described previously^40^. Furthermore, the numbers of internalized Δ*greA* mutant bacteria were 28-fold and 40-fold lower at MOIs of 100:1 and 1000:1, respectively, than those of wild-type U112 strain. *In trans* complementation with the mutated *greA* gene (D43A/E46A) did not rescue the invasion defect of the Δ*greA* mutant, in contrast to complementation with the intact *greA* gene (Fig. 5E). Taken together, these results confirm that *greA* is essential for the efficient entry into and replication within the host cells.

### Loss of GreA influences the expression of various *Francisella* virulence genes

**Figure 6:**
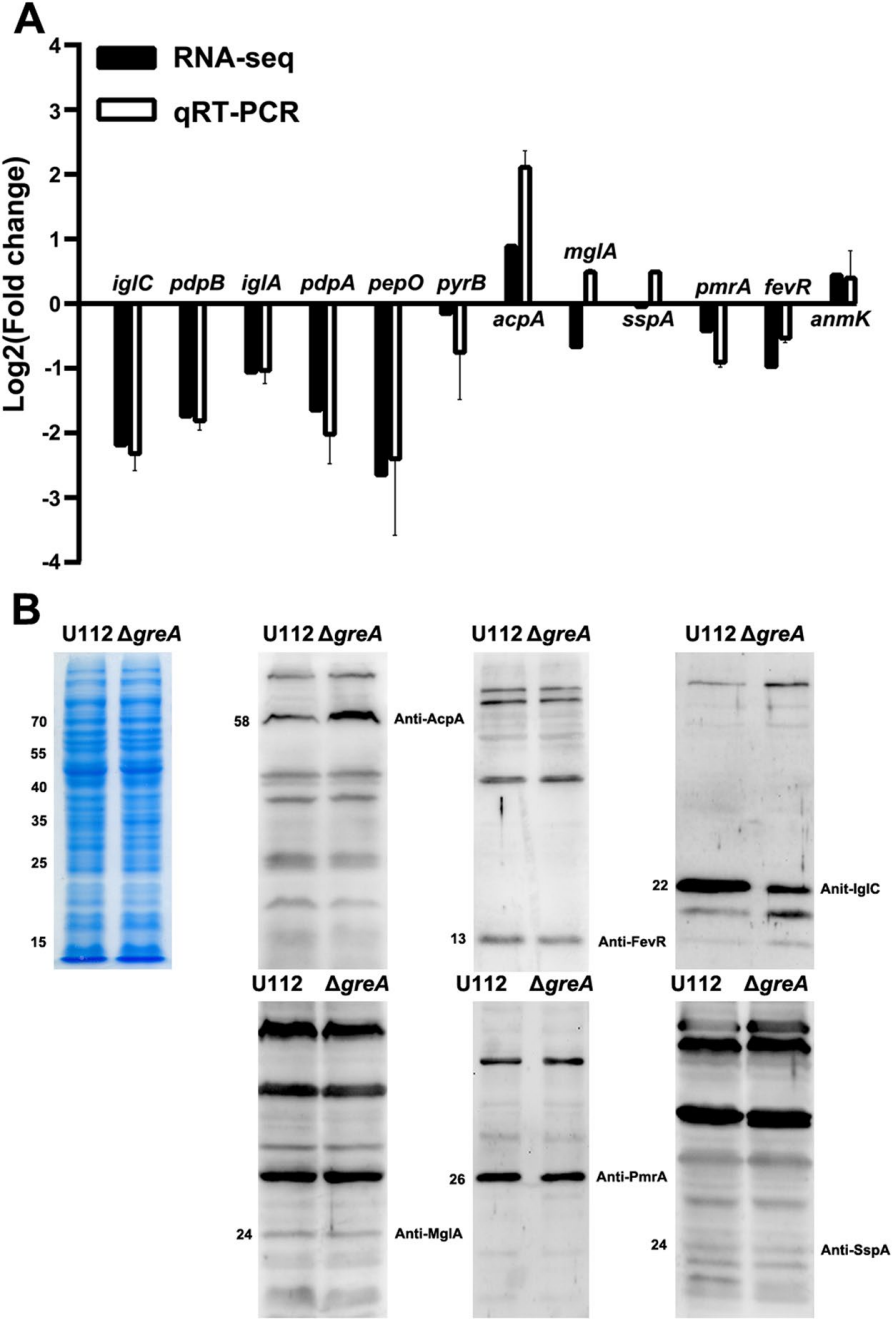
Verification of RNA-seq data. (**A**) Detection of gene transcription in the wild-type U112 strain and the Δ*greA* mutant. Transcription levels of genes by RNA-seq were shown with solid bars. Relative level of each target gene (open bars) by qRT-PCR was normalized to that of the 16S rRNA gene. Data are presented as mean fold changes relative to the wild-type U112 strain ± SD of the results from triplicate samples. The experiment was repeated twice. (**B**) Detection of protein expression in the wild-type U112 strain and the Δ*greA* mutant. Mid-log bacteria were resuspended in PBS to OD_600_ = 1.0. The suspensions were concentrated 10-fold, separated with SDS-PAGE, and detected with western blotting, using antiserum specific for each target protein. The left panel is a section of a coomassie stained gel as a loading control. The coomassie stained gel image was acquired with the digital camera (Canon, Janpan). The experiment was repeated twice. Size of each protein is indicated on the left in kDa.

Because it is a strongly conserved regulatory protein, GreA affects the expression of a variety of genes *in vivo*^24,41^. To clarify its attenuation, we compared the gene expression profiles of *F. novicida* U112 and its isogenic Δ*greA* mutant with an RNA-seq analysis. During the exponential phase of growth, 196 genes showed significant differential expression (by at least 2-fold increase or decrease) compared with their expression in wild-type strain U112 (Table S2, Fig. S2), representing approximately 11% of the *F. novicida* coding genes. Of these genes, 121 were downregulated and 75 were upregulated, and the genes were distributed in multiple functional categories (Table S2, Fig. S2). The RNA-seq analysis results were confirmed with qRT-PCR and western blotting of selected genes (Fig. 6A,B).

It is noteworthy that 77 of the 196 differentially expressed genes have previously been identified as virulence factors of *F. tularensis* with high-throughput genetic screening of mammal and/or arthropod vector models (Table S3)^8,33,42–54^. Among these genes, the first large group is involved in cell metabolism and most of them participate in amino acid biosynthetic pathways involving the utilization of host amino acids (*gcvP1*, *gcvP2*, *gcvH*, and *aroE*) (Table S2). These genes have been identified to be associated with the ability of *F. tularensis* to replicate and survive in the mice^47,52,53^. It is noteworthy that all the genes in the FPI, except *anmK*, *iglF* and *pdpE*, were downregulated (Table S2). However, the transcription of genes known to encode FPI regulator proteins, including *mglA*, *sspA*, *fevR*, and *pmrA*, was not significantly altered in the *greA* deletion mutant relative to the wild-type U112 strain in either the RNA-Seq or qRT-PCR analysis (Fig. 6A). These results suggest that GreA is required for the proper expression of the FPI genes. Although the functional mechanism is not yet clear, the genes in the two operons in FPI responds to GreA in the same way, suggesting that GreA regulates transcription initiation and/or promoter escape, as reported in *E. coli*^41^.

### GreA affects the expression of invasion-associated genes in *F. novicida*

**Figure 7:**
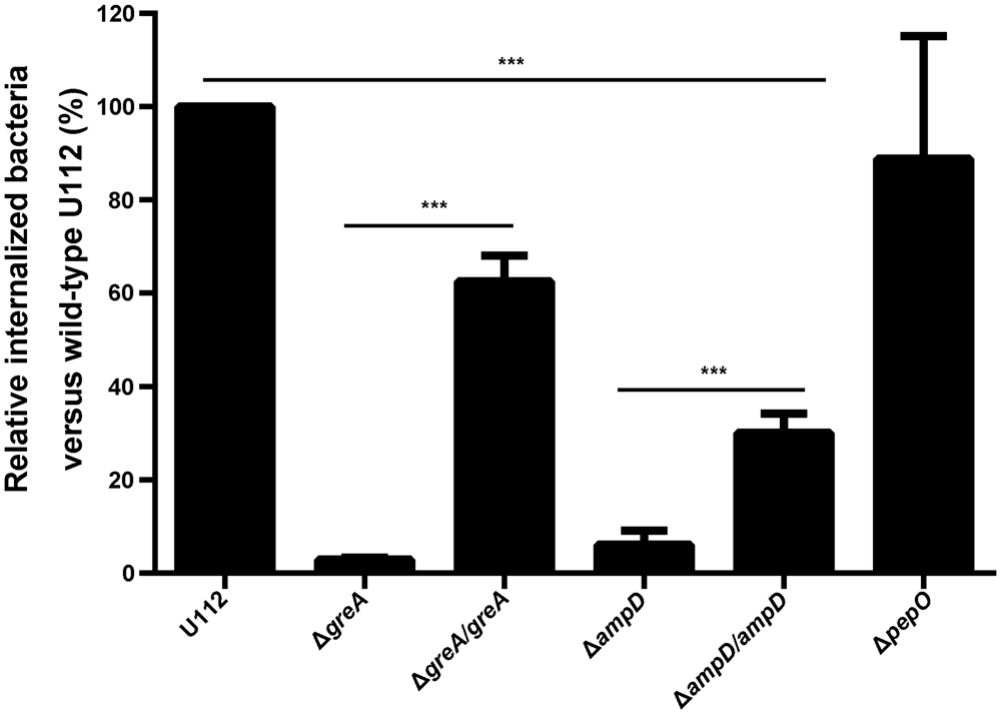
Impact of deletion of *ampD* and *pepO* on invasion of cells by *F. novicida in vitro*. RAW264.7 cells were infected with the wild-type U112 strain or its derivatives at an MOI of 100:1 for 2 h. After treatment with 100 μg/ml gentamycin for 1 h, the infected cells were lysed and spread on TSA plates for enumeration. The values are mean percent of internalized bacteria relative to the wild-type U112 strain ± SD of the results from triplicate samples as compared with those of the wild-type U112 strain. The experiment was repeated twice. Statistical significance was determined by one-way analysis of variance (ANOVA) test. ***p < 0.001.

Based on the finding that the deletion of *greA* impairs the entry of *F. novicida* into its host cells, we hypothesized that the expression of bacterial adhesion/invasion factors are regulated by GreA. To explore this possibility, we selected *ampD* and *pepO* from the downregulated genes (Table S4) because these genes have been shown to be involved in the cellular adhesion and invasion in *Salmonella typhimurium* and *Porphyromonas gingivalis*^55,56^. The Δ*ampD* and Δ*pepO* gene deletion mutants were constructed separately and invasion experiments were performed by infecting RAW267.4 cells. The quantitative analysis of the internalized bacteria 3 h after infection showed that the invasion ability of the Δ*ampD* mutant was reduced compared with that of the wild-type U112 strain, whereas the invasiveness of Δ*pepO* was not significantly different from that of the wild-type U112 (Fig. 7). The *in trans* complementation of the *ampD* gene significantly restored the invasion ability of the mutant (Fig. 7). These results suggest that *ampD* contributes to the entry of *F. novicida* into its host cells, confirming that GreA regulates the expression of cell-invasion-associated genes.

## Discussion

*Francisella tularensis* has developed complex gene expression regulation strategies to finely control the expression of each individual gene in response to its changing environment, but only a few transcriptional regulators and their effects on the expression of virulence factors have been studied^9^. In this study, we have demonstrated the important role of the transcription elongation factor GreA in the pathogenesis of *F. novicida* with the following results: (i) the disruption of the *greA* gene significantly attenuated the virulence of *F. novicida*, impairing its ability to invade and replicate within host cells; (ii) the loss of GreA resulted in significant changes in the expression of 196 genes, including 77 genes that have previously been implicated in the virulence of *F*. *tularensis*; and (iii) *ampD* contributed to the entry of *F. novicida* into its host cells.

We evaluated the importance of GreA in the pathogenicity of *F. novicida* in mice and observed that the deletion of the *greA* gene led to the severe attenuation of *F. novicida* infection in both subcutaneous and intranasal infection models. This was supported by the significantly lower bacterial burdens in the livers, spleens, and lungs of the mice infected with the Δ*greA* mutant, despite the much higher dose of inoculum used, and supports the previous finding that transposon-insertion mutants of the *F. tularensis* LVS *greA* gene failed to grow in mouse lung tissues after intranasal infection^33^.

Our cell culture infection experiments demonstrated that the deletion of the *greA* gene significantly reduced the ability of *F. tularensis* to invade and replicate in both macrophages and epithelial cells, indicating that GreA plays an important role in the internalization and replication phases of infection. Similar results have been reported for *Salmonella typhimurium*, in which the Gre factors were shown to regulate *hilD* expression, playing an important role in the epithelial cell invasion and dissemination of the bacterium *in vivo*^57^.

RNA polymerase backtracking plays important roles in the processes of bacterial gene transcription and protein translation by fine-tuning the expression of genes in response to nutrient availability and environmental cues^58^. One important function of GreA is to rescue backtracking-mediated pauses in transcription that might compromise gene expression, and explains why the deletion of the gene encoding an anti-backtracking factor caused the differential expression of 196 genes involved in multiple functions in the mutant *F. novicida*. A large proportion of the genes downregulated in the *F. novicida greA* mutant are involved major metabolic pathways (Table S2), so this mutation affects the efficiency of nutrient acquisition and metabolism. Notably, more than 26 of these metabolism-related genes have previously been shown or suggested to be virulence factors (Table S3). The *gcvH* and *gcvP1* genes were significantly downregulated. These genes encode enzymes involved in the glycine cleavage system and mutations in these genes reduce intracellular growth or attenuate bacterial virulence in mice^47,51,52^. The products encoded by *fba* and *tktA* contribute to the glycolytic and gluconeogenic pathways of *Francisella*^59^, and are essential for bacterial virulence.

GreA has a role in the cell stress response in other bacteria^25–30^. We observed a growth defect in the Δ*greA* mutant in medium supplemented with 2% NaCl. However, it was attributable to an ionic effect rather than an osmotic effect. The probable explanation is that the inactivation of *greA* affects the function of bacterial chlorine pump (s), because the expression of the *clcA* gene was downregulated in the mutant. This gene encodes ClcA, a component of the H^+^/Cl^−^ antiporters, which is essential for bacterial survival in chlorine-rich environments and has been shown to play an important role in ion homeostasis^60–62^.

Biofilm formation increase was observed in the Δ*greA* mutant. This phenotype was also observed in the *F. novicida hfq* mutant, although it was only detectable in more restrictive MH broth^63^. Chambers and Bender attributed the increased biofilm formation to the upregulation of an outer membrane protein and several type-IV-pili-associated proteins^63^. Zogaj *et al*. identified a biofilm formation signaling cascade in *F. novicida* that included QseB, CdgB, cdGMP, and ChiB, and confirmed that biofilm formation was directly associated with these proteins and molecules^38^. We speculate that the proteins and molecules described above were upregulated in the Δ*greA* mutant during biofilm formation since the transcription of *chiB* increased significantly from 24 h post incubation (Fig. S3), but the regulating mechanism still needs to be investigated.

Our data suggest that GreA regulates the expression of virulence factors associated with bacterial adhesion and invasion. AmpD is a cytosolic *N*-acetylmuramyl-l-alanine amidase that uses recycled muropeptides for peptidoglycan synthesis. The *ampD* mutant of *Salmonella typhimurium* displayed substantially reduced entry into mouse macrophages^56^. Consistent with this, a similar entry defect in the *ampD* mutant was detected in *F. tularensis*. Thus, the downregulation of *ampD* is evidence of invasion impairment in the Δ*greA* mutant.

Interestingly, the expression of the 15 FPI genes was downregulated in the absence GreA, but no significant change was observed in the genes encoding proteins that regulate the expression of FPI genes, such as MglA, SspA, FevR, and PmrA^11,12,14,64^. FPI is a ~30-kb gene cluster that contains two putative divergent operons, and we speculate that GreA stimulates RNAP promoter escape and the transition from the initiation to the elongation stage, as reported in *E. coli*^41^. Furthermore, as shown in previous studies, MglA, SspA, and FevR regulated the similar set of genes^12,64^, but most genes regulated by PmrA did not overlap with those regulated by MglA although most FPI genes were regulated by these regulators^14^. Similarly, most genes regulated by GreA did not overlap with those regulated by MglA or PmrA, which indicated that GreA did not interact with these known regulators. Further investigation is required because we cannot rule out the possible involvement of indirect regulation, with the expression of an unidentified regulator affected.

This study provides further evidence that the transcriptional elongation factor GreA plays an important role in the pathogenesis of *Francisella* by globally regulating the expression of virulence genes and subsequently bacterial entry into and replication within its host cells. The conserved protein GreA is a potential treatment target for new drug design, and the molecular interplay between GreA and virulence-related gene expression offers a new field of investigation.

## Methods

### Bacterial culture, plasmids, chemicals, and primers

The bacterial strains, plasmids, and primers used in this study are listed in Table S1. *Francisella novicida* U112 and its derivatives were cultured at 37 °C in TSB supplemented with 0.1% l-cysteine or on TSA with the same supplement. *Escherichia coli* was grown in Luria–Bertani (LB) broth or on LB agar. When necessary, ampicillin (100 μg/ml), kanamycin (10 μg/ml), or hygromycin (200 μg/ml) was added for selection. To measure the growth kinetics of *F. novicida* strain U112 and its derivatives, the strains were individually cultured overnight in 10 ml of TSB at 37 °C. The strains were then diluted by approximately 0.001 (to OD_600_) in 100 ml of fresh TSB at 37 °C. The growth kinetics were monitored by measuring OD_600_ at the indicated time points. To count the viable bacteria, the bacterial cultures were diluted and spread onto TSA plates at the indicated time points, as described previously^65^. The doubling times were calculated with the equation g = (t − t_0_) × log2/(logA_t_ − logA_0_), where g is the mean doubling time, t and t_0_ are the two time points, A_t_ is the bacterial CFU at time point t, and A_0_ is the bacterial CFU at time point t_0_^66^.

### Antibody generation and western blotting

Briefly, the *greA* open reading frame was amplified from the *F. novicida* U112 genomic DNA with the primer pair Pr0023/Pr0024 (Table S1), and cloned into the plasmid pET-32a (Novagen, Madison, WI, USA). *Escherichia coli* BL21 (DE3) was transformed with the resulting plasmid to express the protein, which was purified with Ni–Agarose (Solarbio, Beijing, China). To produce antiserum, 100 μg of purified recombinant GreA protein in 100 μl of phosphate-buffered saline (PBS) was emulsified with an equal volume of complete Freund’s adjuvant (Sigma-Aldrich, St. Louis, MO, USA) and injected subcutaneously into five BALB/c mice (Charles River Laboratory, Beijing, China). The mice were boosted twice at 2-weekly intervals with the same amount of protein emulsified with incomplete Freund’s adjuvant (Sigma-Aldrich). Two weeks after the last immunization, the mice were anesthetized to collect blood from the venous sinuses of their eyes. Sera were collect and stored at −80 °C. Recombinant FevR, PmrA, SspA, MglA, AcpA, and IglC proteins were individually expressed in a similar manner and the corresponding antisera were produced in mice. The primer pairs used for gene amplification are listed in Table S1.

Western blotting was performed as previously described^65^. Antibodies directed against FevR, PmrA, SspA, MglA, AcpA, GreA, and IglC were used at dilutions of 1:100. Horseradish-peroxide-conjugated goat anti-mouse secondary antibody (ZSGB-Bio, Beijing, China) was used at a dilution of 1:5000. The antibodies were detected with ECL reagent (CWBIO, Beijing, China) by the automatic chemiluminescence image analysis system (Tanon, Shanghai, China).

### Construction of *F. novicida* deletion mutant and complemented strain

The *greA* deletion mutant was constructed using a previously described method^67^. Briefly, fragments up- and downstream from the *greA* gene were amplified with PCR from the genomic DNA of *F. novicida* U112 with primer pairs Pr0001/Pr0002 and Pr0003/Pr0004, respectively, and the kan^r^ cassette was amplified from the plasmid pMOD2EZTN-FT_Kan^r^ with primer pairs Pr0019/Pr0020 (Table S1). The three amplicons were then joined with overlapping PCR and wild-type U112 cells were transformed with the fusion construct using electroporation. After incubation on TSA agar plates with kanamycin for 24 h, the transformants were picked and the deletion of the target gene was confirmed with PCR and a western blot analysis using mouse antiserum directed against GreA. The *ampD* and *pepO* deletion mutants were similarly generated.

To construct the *greA* mutant complementation strain, the intact *greA* gene was amplified from the *F. novicida* U112 genome with primer pair Pr0021/Pr0022 (Table S1) and cloned into pMP633, as previously described^33,68^. The Δ*greA* mutant was transformed with the recombinant plasmid with electroporation, generating the *in trans-*complemented strain Δ*greA*/*greA*. The *ampD* mutant complementation was similarly constructed.

As a control, the active sites of GreA, the aspartic acid at amino acid 43 (D43) and the glutamic acid at amino acid 46 (E46), were converted to alanine (A) with PCR, using primer pairs Pr0021/Pr0100 and Pr0022/Pr0101, respectively (Table S1), and the *in trans*-complemented strain with the mutated *greA* gene (Δ*greA*/*greA* D43A/E46A) was constructed as described above.

### Transcriptional analysis of genes in the *greA* cluster

*Francisella novicida* U112 and the Δ*greA* mutant were cultured to mid-log phase (OD_600_ = 1.0) in TSB. The bacterial genomic DNAs were extracted from the cultures with the TIANamp Bacteria DNA Kit (Tiangen, Beijing, China) and quantified with a NanoDrop 2000 spectrophotometer (Thermo Scientific, Waltham, MA, USA). The total bacterial RNAs were isolated from cultures with TRIzol Reagent (Ambion, Austin, TX, USA) and digested with DNase I (NEB, Ipswich, England) to eliminate any genomic DNA contamination.

To determine whether these seven genes (*pepA*, *guaB*, FTN_0662, *pgi*, *fimT*, *greA*, and *uvrA*) were cotranscribed, the junction regions were amplified with RT–PCR. The intergenic cDNAs were synthesized from the extracted *F. novicida* RNA with the GoScript™ Reverse Transcription System (Promega, Madison, WI, USA) and primers Pr0038, Pr0040, Pr0042, Pr0044, Pr0046, and Pr0048, respectively (Table S1). The junction PCRs were performed with the synthesized cDNA templates and the primer pairs indicated in Fig. 1B and Table S1. A DNA-free RNA extract and the genomic DNA of *F. novicida* were used as the controls.

To determine whether the deletion of *greA* affected the transcription of other genes (*pepA*, *guaB*, FTN_0662, *pgi*, *fimT*, and *uvrA*) in this operon, the transcription of these genes was detected with qRT-PCR, as described previously^65^. The cDNAs were synthesized from the bacterial RNAs described above with random primers, using the GoScript™ Reverse Transcription System (Promega). The qRT-PCR assays were performed with the LightCycler® 480 System (Roche, Basel, Switzerland) with SuperReal PreMix Plus (SYBR Green) (Tiangen), according to the supplier’s instructions. The primers used for qRT-PCR are listed in Table S1. All reactions were performed in triplicate, in three independent assays. The transcript levels of the target genes were normalized to the levels of 16S rRNA and analyzed with the 2^−ΔΔCT^ method^69^.

### Detection of bacterial stress tolerance and biofilm formation

Wild-type strain U112 and its derivatives were cultured in TSB overnight at 37 °C with rotation (160 r/min) and their concentrations adjusted to OD_600_ = 1.0. The cultures were further diluted 1000-fold in TSB, with or without 2% NaCl, and the diluted suspensions were dispensed into sterile 96-well plates (200 μl/well) for analysis with the Bioscreen-C™ Automated Microbiology Growth Curve Analysis System (Bioscreen, Finland). The plates were incubated at 37 °C with intermediate speed and the growth data were recorded at 30 min intervals for 48 h.

The biofilms formed by the wild-type strain U112 and its isogenic mutants were detected as previously described^70^. Briefly, 96-well sterile plates were inoculated with 100-fold-diluted mid-log bacteria (200 μl/well) and incubated at 37 °C. At 48, 72, and 96 h post inoculation, the OD of the cultures at 630 nm (OD_630_) was measured with a microplate reader (Thermo Scientific). The culture supernatants were removed by aspiration and the biofilms were washed twice with PBS, fixed with 99% methanol for 15 min, and stained with crystal violet for 5 min. The wells were washed with water and dried. The dye was redissolved with 33% acetic acid and the OD of the dye solution was measured at 570 nm (OD_570_).

### Infection of cell cultures

Mouse bone-marrow-derived macrophages were prepared from BALB/c mice (Charles River Laboratories), as previously described^71^. RAW264.7 cells and A549 cells were purchased from the China Infrastructure of Cell Line Resources (Beijing, China). The cells were cultured in RPMI 1640 (Gibco, Grand island, NY, USA) supplemented with 10% fetal bovine serum (Gibco) and 25 mM HEPES at 37 °C under 5% CO_2_. The cells were grown in 24-well plates to approximately 80% confluence before infection. The mid-log bacteria described above were diluted to OD_600_ = ~0.3 in prewarmed RPMI 1640. The cells were infected at an MOI of 100 bacteria per cell (0 h). The bacteria and cells were cocultured for 2 h to allow the bacteria to invade the cells, and the samples were then treated with 100 μg/ml gentamycin for 1 h to kill the extracellular bacteria^72^. At 3–24 h post inoculation a lower concentration of gentamycin (10 μg/ml) was applied to prevent the growth of extracellular bacteria. At the indicated time points, the bacteria were released by lysing the cells from triplicate wells with chilled water. The lysates were serially diluted and plated on TSA for enumeration.

To quantify bacterial invasion, RAW264.7 cells were infected with the wild-type U112 strain and its derivatives at the indicated MOIs. The samples were treated with 100 μg/ml gentamycin for 1 h after the bacteria and cells were cocultured for 2 h. At 3 h post infection, the infected cells were lysed to count the CFU of the invasive bacteria.

To quantify bacterial adhesion, RAW264.7 cells were pretreated with 1 μg/ml cytochalasin D (Sigma-Aldrich) for 1 h to block the internalization of the bacteria, as previously described^65^. The cells were then infected with wild-type strain U112, the Δ*greA* mutant, or the complemented strain at the indicated MOIs. After incubation at 37 °C for 2 h, the infected cells were lysed to count the CFU of the adherent bacteria.

### Mouse infections

To determine the pathogenicity of *F. novicida* strain U112 and its derivatives, groups of five BALB/c mice (female, 6–8 weeks old; Charles River Laboratories) were subcutaneously or intranasally inoculated with different doses of wild-type strain U112, the Δ*greA* mutant, or the complemented strain. The signs of morbidity and mortality in the challenged mice were monitored daily for 21 days.

To test the effect of the deletion of the *greA* gene on bacterial dissemination in mouse organs, groups of five mice were subcutaneously infected with the wild-type U112 strain (1 × 10^3^ CFU/mouse), the Δ*greA* mutant (3 × 10^5^ CFU/mouse), or Δ*greA*/*greA* (1 × 10^3^ CFU/mouse). At 2, 4, 6, and 8 days post infection, five mice per group were killed and their livers, spleens, and lungs were removed, homogenized, and plated on TSA plates for CFU counting.

### RNA-seq

Overnight cultures of wild-type strain U112 and the Δ*greA* mutant were diluted 100-fold with fresh TSB and cultured in triplicate to mid-log phase on a shaker. Total RNA of each sample was extracted using TRIzol Reagent (Invitrogen, Carlsbad, CA, USA)/RNeasy Mini Kit (Qiagen, Dusseldorf, Germany). The prepared RNAs were analyzed with a NanoDrop 2000 spectrophotometer (Thermo Scientific) and Agilent 2100 Bioanalyzer (Agilent, San Diego, CA, USA), and purified with the RNeasy MinElute Cleanup Kit (Qiagen). The Ribo-Zero™ Magnetic Kit (Epicentre, Madison, WI, USA) was used to remove the rRNA, and the rRNA-depleted RNA was used to generate cDNA libraries with the NEBNext Ultra™ Directional RNA Library Prep Kit for Illumina® (NEB). The cDNA was sequenced with the Illumina sequencing technology (Illumina HiSeq™ 2500).

The raw reads were processed with the Cutadapt (version 1.9.1) software. The clean reads were evaluated with the FastQC software (version 0.10.1) and were mapped to the *F. tularensis* subsp. *novicida* genome (NC_008601) with Bowtie 2 (version 2.1.0). In the beginning transcripts in fasta format are converted known gff annotation file and indexed properly. Then, with the file as a reference gene file, HTseq (v0.6.1p1) estimated gene expression levels from the pair-end clean data. The genes differentially expressed between wild-type strain U112 and the Δ*greA* mutant were identified with a model based on the negative binomial distribution in the DEGseq package^73^. After adjusted by Benjamini and Hochberg’s approach for controlling the false discovery rate (FDR < 0.05), *p*-value of genes were set <0.05 and a fold change of >2 was used as the cut-off values to define significantly differential expression between the two strains.

The differentially expressed genes were categorized by their Clusters of Orthologous Groups (COG) annotations^74^. The FPI genes were sorted into a separate class. Genes with no COG annotation were assigned to the ‘poorly characterized’ category.

To verify the results of RNA-seq, qRT-PCR and western blotting assays were performed as previously described^65^. The qRT–PCR assays were performed with the cDNA described above and the primer pairs listed in Table S1, as previously described. For western blotting, mid-log phase cultures were washed twice with sterile PBS and resuspended in PBS to an OD_600_ = 1.0. An aliquot (1 ml) of each suspension was resuspended in 100 μl of PBS and protein loading buffer was added. The samples (10 μl) were separated with sodium dodecyl sulfate-polyacrylamide gel electrophoresis (SDS-PAGE) and analyzed with western blotting.

### Ethics statement

Animal experiments were conducted under protocols approved by the China Agricultural University Animal Ethics Committee, in accordance with the guidelines of the Review of Welfare and Ethics of Laboratory Animals approved by the Beijing Municipality Administration Office of Laboratory Animals.

### Statistical analysis

All graphs were drawn with Graphpad Prsim 6 software. All images and graphs were processed by Adobe Photoshop CC2018 software. The results of representative experiments are shown as means ± standard deviations of the means (SD). Statistical significance of the data from qRT-PCR and doubling time experiments was determined by two-tailed unpaired Student’s *t* test. The data from animal infection, cell experiments, and biofilm experiments were analyzed by one-way analysis of variance (ANOVA) test, respectively. Significant differences are defined by *p* values < 0.05 (*), <0.01 (**), and <0.001 (***).

### Data availability

The datasets generated during and/or analysed during the current study are available from the corresponding author on reasonable request.

## Electronic supplementary material

Figure S1

Figure S2

Figure S3

Table S1

Table S2

Table S3

Table S4
