# Supplementary material for: Transcription Elongation Factor GreA Plays a Key Role in Cellular Invasion and Virulence of Francisella tularensis subsp. novicida
Source: Sci Rep. 2018 May 2;8:6895. doi: 10.1038/s41598-018-25271-5 (PMC5932009; doi:10.1038/s41598-018-25271-5)
Supplement: Supplementary file 1 — Figure S1 [file 41598_2018_25271_MOESM1_ESM.pdf]

Transcription Elongation Factor GreA Plays a Key Role in Cellular Invasion and Virulence of *Francisella tularensis* subsp. *novicida*

Guolin Cui[1], Jun Wang[1], Xinyi Qi[1], Jingliang Su[1*]

Supplementary Figure S1

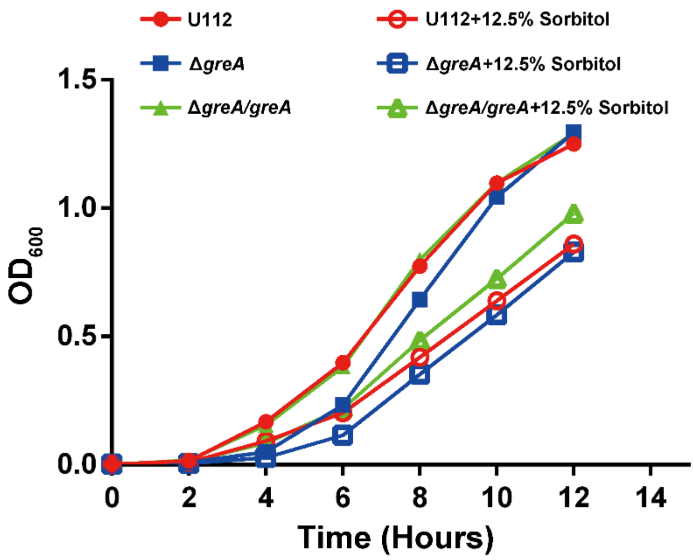

Figure S1. Osmotic tolerance of *F. novicida*.
Overnight cultures of the wild-type U112 strain and its derivatives were adjusted to OD_{600}=1.0. The cultures were diluted 1000-fold in TSB, with or without 12.5% sorbital, and the diluted suspensions were cultured in a shaker at 37 °C. At indicated time points, the OD_{600} of cultures was detected. The values represent the means ± SD (n=3) from one of the three independent experiments.
