# Supplementary material for: Transcription Elongation Factor GreA Plays a Key Role in Cellular Invasion and Virulence of Francisella tularensis subsp. novicida
Source: Sci Rep. 2018 May 2;8:6895. doi: 10.1038/s41598-018-25271-5 (PMC5932009; doi:10.1038/s41598-018-25271-5)
Supplement: Supplementary file 2 — Figure S2 [file 41598_2018_25271_MOESM2_ESM.pdf]

Transcription Elongation Factor GreA Plays a Key Role in Cellular Invasion and Virulence of *Francisella tularensis* subsp. *novicida*

Guolin Cui[1], Jun Wang[1], Xinyi Qi[1], Jingliang Su[1*]

Supplementary Figure S2

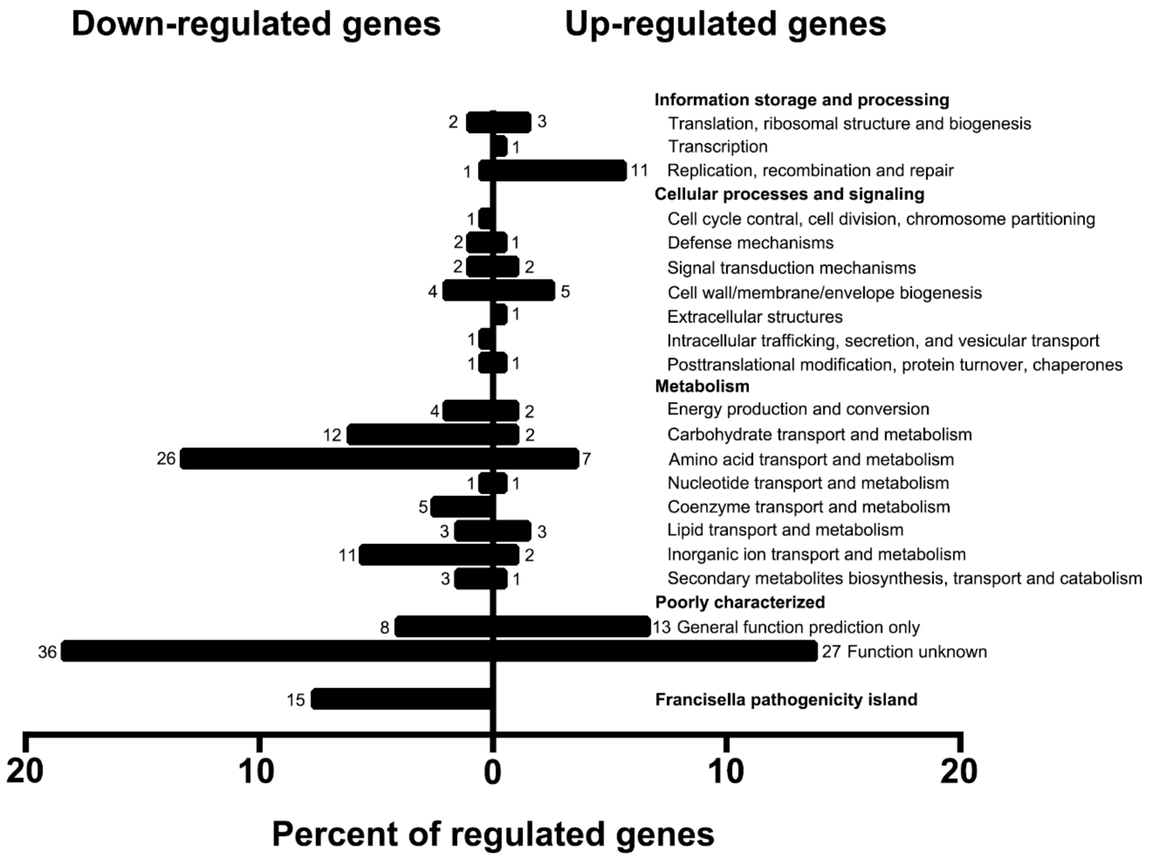

Figure S2. Functional classification of differentially expressed genes.
Gene functions are sorted based on COG categories. The numbers of differently functional genes were marked on the top of the column, respectively.
