# Supplementary material for: Transcription Elongation Factor GreA Plays a Key Role in Cellular Invasion and Virulence of Francisella tularensis subsp. novicida
Source: Sci Rep. 2018 May 2;8:6895. doi: 10.1038/s41598-018-25271-5 (PMC5932009; doi:10.1038/s41598-018-25271-5)
Supplement: Supplementary file 3 — Figure S3 [file 41598_2018_25271_MOESM3_ESM.pdf]

Transcription Elongation Factor GreA Plays a Key Role in Cellular Invasion and Virulence of *Francisella tularensis* subsp. *novicida*

Guolin Cui[1], Jun Wang[1], Xinyi Qi[1], Jingliang Su[1*]

Supplementary Figure S3

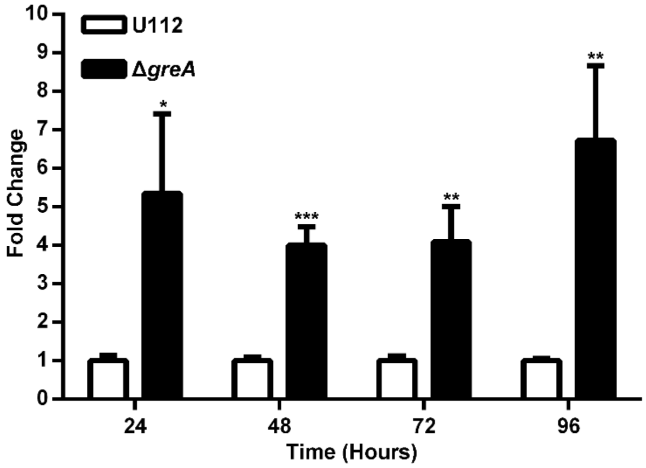

Figure S3. Transcription of *chiB* gene during biofilm formation in wild-type strain U112 and the Δ*greA* mutant.

The 96-well sterile plates were inoculated with 100-fold-diluted mid-log bacteria (200 μl/well) and incubated at 37 °C. At 24, 48, 72, and 96 h, supernatant cultures were removed and TRIzol reagent (200 μl/well) was added to extract bacterial total RNA. Data are the relative transcription levels of each target gene normalized to that of the 16S rRNA gene. Results are shown as the mean fold changes relative to the wild-type U112 strain ± SD (n = 3) from one of the three independent experiments. Statistical significance was determined with unpaired Student's *t* test.
