# Supplementary material for: Transcription Elongation Factor GreA Plays a Key Role in Cellular Invasion and Virulence of Francisella tularensis subsp. novicida
Source: Sci Rep. 2018 May 2;8:6895. doi: 10.1038/s41598-018-25271-5 (PMC5932009; doi:10.1038/s41598-018-25271-5)
Supplement: Supplementary file 4 — Table S1 [file 41598_2018_25271_MOESM4_ESM.pdf]

Transcription Elongation Factor GreA Plays a Key Role in Cellular Invasion and Virulence of *Francisella tularensis* subsp. *novicida*

Guolin Cui[1], Jun Wang[1], Xinyi Qi[1], Jingliang Su[1*]

Table S1 Strains, plasmids, and primers used in this study

Strains, plasmids, and primers	Description	Source or reference
**Strains**		
U112	Wild-type *Francisella tularensis* subspecies *novicida*	Jingren Zhang
Δ*greA*	U112 with deletion in *greA* gene, where Kan^r cassette inserts	This study
Δ*pepO*	U112 with deletion in *pepO* gene, where Kan^r cassette inserts	This study
Δ*ampD*	U112 with deletion in *ampD* gene, where Kan^r cassette inserts	This study
Δ*ampD/ampD*	Δ*ampD* mutant complemented with the integrating pMP633::*ampD* construct	
Δ*greA/greA*	Δ*greA* mutant complemented with the integrating pMP633::*greA* construct	This study
Δ*greA*/pMP633	Δ*greA* mutant complemented with the pMP633 plasmid	This study
Δ*greA/greA* (D43A/E46A)	Δ*greA* mutant complemented with the integrating pMP633::*greA* (D43A/E46A) construct	This study
Escherichia coli DH 5α	F-φ80 lac ZΔM15 Δ(lacZYA-arg F) U169 endA1 recA1 hsdR17(rk-,mk+) supE44λ- thi -1 gyrA96 relA1 phoA	TansGen Biotech

Escherichia coli BL21 (DE3)	F- ompT hsdS(rB - mB -) gal dcm(DE3)	TansGen Biotech
**Plasmids**		
pMP633	A shuttle plasmid for *Francisella*, hyg^r	[1]
pMP633::*ampD*	An integrating complementation vector carrying the *ampD* locus, hyg^r	
pMP633::*greA*	An integrating complementation vector carrying the *greA* locus, hyg^r	This study
pMP633::*greA* (D43A/E46A)	An integrating complementation vector carrying the *greA* locus in which the 43rd aspartic acid (D) and 46th glutamic acid (E) were substituted with alanine (A), hyg^r	This study
pMOD2EZTN-FT-Km^r	An modified vector containing Kan^r cassette expressing in *F. tularensis* or *E. coli*, Kan^r	Jingren Zhang
pET-32a	*E. coli* expression vector	Novagen
**Mutant primers**		
Pr0001 (*greA*)	TTTGTTCAGGGTGTATTG	
Pr0002 (*greA*)	CATGCAAGCTTGCCAACGACTACTGCTGGAGTCATAGGTAC	
Pr0003 (*greA*)	TTCGAGCCAATATGCGAGAACATCTGTTGTTGCTCCTTTA	
Pr0004 (*greA*)	TATTGATTCAGCGATACG	
Pr0007 (*ampD*)	AGCTTTAGACCATAAATATGT	

Pr0008 (*ampD*)	CATGCAAGCTTGCCAACGACTATATACCAGCCTTGATTAAACA
Pr0009 (*ampD*)	TTCGAGCCAATATGCGAGAACACTGATCCTGGTAAATGTTTTGAATGG
Pr0010 (*ampD*)	GGTTCTGCTAAACAAGCACGT
Pr0015 (*pepO*)	GTAGATAAGCTTAATCTTGCA
Pr0016 (*pepO*)	CATGCAAGCTTGCCAACGACTAAACACAGCTAAAATCAAACAGA
Pr0017 (*pepO*)	TTCGAGCCAATATGCGAGAACATTGGAGATGGAATGTACTTAGATCCA
Pr0018 (*pepO*)	CTTGCCGCAACTGAAGCTG
Pr0019 (kan^r cassette)	TAGTCGTTGGCAAGCTTGCATG
Pr0020 (kan^r cassette)	TGTTCTCGCATATTGGCTCGAA
Pr0021 (*greA* complementation)	AGACATATGGCAAATGATAGAGTACCTATGACTCC
Pr0022 (*greA* complementation)	TTGTGTCATATGAAAACTCCTTTAAATACAGCCACTTAATCCTGTGATAACAGT
Pr0100 (*greA* D43A/E46A)	ATTTGCTTTTAGAGCGCCATGATCACGAGCC

Pr0101 (*greA* D43A/E46A)	GGCTCGTGATCATGGCGCTCTAAAAGCAAAT
Pr0104 (*ampD* complementation)	AGACATATGTTTAATCAAGGCTGGTA
Pr0105 (*ampD* complementation)	CTTGTGTCATATGAAAACTCCTTTAAATACTACCAGATAACCTTATTCCATTCA
**Protein expression primers**	
Pr0023 (GreA)	GGGGTACCATGGCAAATGATAGAGTACCT
Pr0024 (GreA)	CGGGATCCTTTATATTCTACAGCTACAATTTCGT
Pr0025 (MglA)	GGGGTACCTTGCTTTTATACACAAAAAAAGATGA
Pr0026 (MglA)	CGGGATCCAGCTCCTTTTGCTTTGATAGT
Pr0027 (IglC)	GGGGTACCATGAGTGAGATGATAACAAGACAACA
Pr0028 (IglC)	CGGGATCCTGCAGCTGCAATATATCCTA
Pr0029 (FevR)	GGGGTACCATGGCGAATCAATATTCTGGA

Pr0030 (FevR)	CGGGATCCAGATTTAGCTTTGATTACAGAA
Pr0031 (SspA)	GGGGTACCTTGATGAAAGTTACATTATATACAACG
Pr0032 (SspA)	CGGGATCCTCTATGAGCTCTTAGAGTTTTGAGT
Pr0033 (PmrA)	GGGGTACCATGAGAATATTGTTGGCTGAAGA
Pr0034 (PmrA)	CGGGATCCCTTAATTACTTTATCCTTTTGTACA
Pr0035 (AcpA)	GGGGTACCATGAAGCTCAATAAAATTACTTTAGGA
Pr0036 (AcpA)	CGGGATCCTTAGTTTAATTTATCCATCACTAATCC
**Junction PCR primers**	
Pr0037 (*pepA-guaB*)	TGCCGATATGGATAACTGTGGT
Pr0038 (*pepA-guaB*)	ACCAATACCACCTTCCTGAGC
Pr0039 (*guaB-FTN_0662*)	AGCTTTTCCAAGGTCGCTCT
Pr0040 (*guaB-FTN_0662*)	TGATAAGCCCCATCAGCACA

Pr0041 (*FTN_0062-pgi*)	GGTATCCTTTAAGCGGCGAGA
Pr0042 (*FTN_0062-pgi*)	CGCAACGCCGTATGTAAAACA
Pr0043 (*pgi-fimT*)	AGTCTTGGTGCCTTGATTGCT
Pr0044 (*pgi-fimT*)	GTCGCTGCGCATACAATAACA
Pr0045 (*fimT-greA*)	TGGAAACAGTTTTGCATGTGCA
Pr0046 (*fimT-greA*)	AAGCTTGTTCTCCTGCTGGA
Pr0047 (*greA-uvrA*)	CTCCTTTAGCTCGCGCTCTA
Pr0048 (*greA-uvrA*)	CTATTGAAATAGCTGGAGAAAGGCC
**qRT-PCR primers**	
Pr0049 (16S rRNA)	ACCGATACTGACACTGA
Pr0050 (16S rRNA)	TTACACCGACTCCAACA
Pr0051 (*mglA*)	CGCTTAGCACAGATGATTTTGC
Pr0052	GCATTAGCTCGCTGTTTTGG

(*mglA*)	
Pr0053 (*sspA*)	TGGCGACTTAGAACCAGCTATG
Pr0054 (*sspA*)	ACATTTGGTAGCAAGCTTGGC
Pr0055 (*pmrA*)	AGCTGTGTCTTTGGTACTACCC
Pr0056 (*pmrA*)	TCCAAGCTCTGAAACGGTAACC
Pr0057 (*iglC*)	AGCTTTCGTGACATCCTCTCC
Pr0058 (*iglC*)	GCGAGACCATTCATGTGAGAAC
Pr0059 (*pdpB*)	AAGTCAGCCATCAACAGAGC
Pr0060 (*pdpB*)	CTTTCGGAGTCTTCACCTACAAC
Pr0061 (*pepO*)	CTCGCCATATTTGTGACCAACC
Pr0062 (*pepO*)	TTGCGGATAATGCAGGCTTG
Pr0063 (*pyrB*)	TGGTTAGTGAGTGAACCGTACG
Pr0064 (*pyrB*)	TGCTGGCGATGGTGAAAATG
Pr0065	TGACCTCTCTATCTGCGAACTC

(*iglA*)	
Pr0066 (*iglA*)	CTTACAGAGTCCTAGTTGTTGGC
Pr0067 (*pdpA*)	CAGCAGCAACTACTACAACGAC
Pr0068 (*pdpA*)	GTCTCGCTATCACTTGTTTGGG
Pr0069 (*acpA*)	GCTGTGCTATCCCAGTCTTTTG
Pr0070 (*acpA*)	AACAACATGCCTGCGGTAAG
Pr0071 (*anmK*)	ACTTTTTGCTCCGGTTGCAG
Pr0072 (*anmK*)	GTGATGCGAAAGAGGCTATAGC
Pr0073 (*fevR*)	AGCTCCCGCCTAGATTTAGATC
Pr0074 (*fevR*)	CCAGCAGCGACTAAAAACATTTC
Pr0075 (*pepA*)	TGGGCGCATCTTGATATTGC
Pr0076 (*pepA*)	AATGGAACCGGTCTACCTGAG
Pr0077 (*guaB*)	ATGAACAAGGCGAGTTGGTC
Pr0078	TGCTGCAACACGCTCTTTAG

(*guaB*)	
Pr0079 (*FTN_0062*)	TGTATTTGGCGCTTCTGAGG
Pr0080 (*FTN_0062*)	CAACACCGCTTACAGCAAAC
Pr0081 (*pgi*)	ATACGGCGTTGCGTGATTTG
Pr0082 (*pgi*)	TTACCAGAGAATCCACGCCATC
Pr0083 (*fimT*)	ATGATGGCGGCAATCAGTTC
Pr0084 (*fimT*)	TGTCGCTGCGCATACAATAAC
Pr0085 (*uvrA*)	AATGTTCGTGGCGGAAGATG
Pr0086 (*uvrA*)	TTGCACCGCTAAAGTTTCGC

References

1 LoVullo, E. D., Sherrill, L. A., Perez, L. L. & Pavelka, M. S., Jr. Genetic tools for highly pathogenic Francisella tularensis subsp. tularensis. *Microbiol* **152**, 3425-3435, doi:10.1099/mic.0.29121-0 (2006).
