# Supplementary material for: Transcription Elongation Factor GreA Plays a Key Role in Cellular Invasion and Virulence of Francisella tularensis subsp. novicida
Source: Sci Rep. 2018 May 2;8:6895. doi: 10.1038/s41598-018-25271-5 (PMC5932009; doi:10.1038/s41598-018-25271-5)
Supplement: Supplementary file 5 — Table S2 [file 41598_2018_25271_MOESM5_ESM.pdf]

Transcription Elongation Factor GreA Plays a Key Role in Cellular Invasion and Virulence of *Francisella tularensis* subsp. *novicida*

Guolin Cui[1], Jun Wang[1], Xinyi Qi[1], Jingliang Su[1*]

Table S2 Genes regulated by GreA in *F. novicida*

Gene number for *F.novicida* U112 (updated by 30-May-2017)	Gene number for *F.novicida* U112	Gene number for *F.tularensis* LVS	Gene number for *F.tularensis* schu S4	Gene name	COG[1]	Pathway or transport system[2]	Gene function	Fold change
				**Information storage and processing**				
FTN_RS00050	FTN_0010	NA	NA		L		Terminase small subunit	4.56
FTN_RS01095	FTN_0213	FTL_0209	FTT_0298	*holC*	L		DNA polymerase III subunit	0.45
FTN_RS01975	FTN_0380	NA	NA		K		Transcriptional regulator	2.60
FTN_RS02415	FTN_0465	FTL_0401	FTT_1293c		J		Threonylcarbamoyl-AMP synthase	2.58
FTN_RS03120	FTN_0603	FTL_1543	FTT_0693c	*mutM*	L		Formamidopyrimidine-DNA glycosylase	2.06
FTN_RS03495	NA	NA	FTT_1583		L		IS1595 family transposase ISFtu6	2.23
FTN_RS03510	FTN_0679	FTL_1449	FTT_0776c		J		Ribonuclease D	2.42
FTN_RS04000	FTN_0776	FTL_1102	FTT_1097		L		ATP-dependent helicase	2.32
FTN_RS04320	FTN_0838	FTL_1244	FTT_0959c		L		Exodeoxyribonuclease III	2.07
FTN_RS05175	FTN_1004	FTL_0955	FTT_0679c	*ychF*	J		Redox-regulated ATPase	2.25
FTN_RS05470	FTN_1063	FTL_0886	FTT_0618c	*miaB*	J		tRNA (N6-isopentenyl	0.43

						adenosine(37)-C2)-methylthiotransferase	
FTN_RS06110	FTN_1191	FTL_1407	FTT_0817		J	Threonine--tRNA ligase	0.42
FTN_RS07265	NA	NA	FTT_1792c		L	IS1595 family transposase ISFtu7	2.70
FTN_RS07820	FTN_1528	FTL_0275	FTT_1518	*ogt*	L	Methylated-DNA--[protein]-cysteine S-methyltransferase	2.36
FTN_RS08255	FTN_1612	FTL_1674	FTT_0103c		L	Transposase	2.14
FTN_RS09255	NA	NA	NA		L	IS1595 family transposase	3.19
FTN_RS09345	NA	NA	NA		L	IS1595 family transposase	2.44
FTN_RS09360	NA	NA	NA		L	IS630 family transposase	3.19
**Cellular processes and signaling**							
FTN_RS00370	FTN_0072	FTL_0179	FTT_0232c	*ddg*	M	Acyltransferase	2.30
FTN_RS00405	FTN_0079	FTL_0172	FTT_0239	*murC*	M	UDP-N-acetylmuramate--L-alanine ligase	2.28
FTN_RS01490	FTN_0289	FTL_1710	FTT_1640c	*proQ*	T	Activator of osmoprotectant transporter ProP	2.54
FTN_RS01510	FTN_0293	FTL_1706	FTT_1636		M	Hypothetical protein	0.45
FTN_RS01515	FTN_0294	FTL_1705	FTT_1635	*ftsK*	D	DNA translocase	0.48
FTN_RS01575	NA	FTL_1692	FTT_1623c		W	Pilus assembly protein	2.08
FTN_RS02240	FTN_0431	FTL_0425	FTT_0905		M	Type IV pili glycosylation protein	0.46
FTN_RS03615	FTN_0700	NA	FTT_0735		MR	Dipeptide epimerase	2.17
FTN_RS03955	FTN_0767	FTL_1091	FTT_1107c	*betT*	M	BCCT family transporter	0.48
FTN_RS04440	FTN_0862	FTL_1218	FTT_0981		T	Diguanylate cyclase	0.40
FTN_RS04840	FTN_0937	NA	NA		T	Hypothetical protein	2.47
FTN_RS05475	FTN_1064	FTL_0885	FTT_0617c	*phoH*	T	PhoH family protein	0.48
FTN_RS05515	FTN_1072	FTL_0879	FTT_0611c		V	FTU family class A beta-lactamase	0.34

FTN_RS05630	FTN_1096	FTL_0847	FTT_1116c	*yajC*	U		Preprotein translocase subunit	0.46
FTN_RS06085	FTN_1186	NA	FTT_1209c	*pepO*	O		M13 family peptidase	0.16
FTN_RS06255	FTN_1220	FTL_1431	FTT_0790		M		Sugar transferase	2.06
FTN_RS06420	FTN_1252	FTL_0710	FTT_1234		M		Linear amide C-N hydrolase	2.14
FTN_RS07190	FTN_1405	FTL_0623	FTT_1435c		V		ABC transporter ATP-binding protein	2.06
FTN_RS07940	FTN_1551	FTL_1727	FTT_0162	*ampD*	V		1,6-anhydro-N-acetylmuramyl-L-alanine amidase	0.33
FTN_RS08870	FTN_1728	NA	NA	*dapA*	ME		Dihydrodipicolinate synthase family protein	0.21
FTN_RS09015	FTN_1756	NA	NA		O		Thioredoxin-dependent thiol peroxidase	2.05
					**Metabolism**			
FTN_RS00020	FTN_0004	NA	NA		E	APC	APC family permease	0.40
FTN_RS00040	FTN_0008	NA	NA		RGE		EamA/RhaT family transporter	0.45
FTN_RS00170	FTN_0033	NA	NA		E		Chorismate mutase	0.39
FTN_RS00400	FTN_0078	FTL_0173	FTT_0238	*aroE*	E	Aromatic amino acid biosynthetic pathway	Shikimate dehydrogenase	2.19
FTN_RS00995	FTN_0194	FTL_0190	FTT_0280c	*yajR*	PRGE	MFS	MFS transporter	0.48
FTN_RS01120	FTN_0217	FTL_0214	FTT_0303c	*lldD*	C		L-lactate dehydrogenase	2.43
FTN_RS01165	FTN_0223	NA	FTT_0310		E	APC	APC family permease	0.38
FTN_RS01400	FTN_0271	FTL_1310	FTT_0373c	*ndk*	F		Nucleoside-diphosphate kinase	3.83
FTN_RS01405	FTN_0272	FTL_1309	FTT_0372c	*accD*	I		Acetyl-CoA carboxylase carboxyltransferase subunit beta	2.01
FTN_RS02050	FTN_0394	NA	FTT_0866c		P		Heavy metal translocating P-type ATPase	0.46

FTN_RS02110	FTN_0405	FTL_0380	FTT_0879	*sodC*	P		Superoxide dismutase	0.30
FTN_RS02115	FTN_0406	FTL_0381	FTT_0880		I		Sterol desaturase family protein	0.46
FTN_RS02505	FTN_0481	FTL_0450	FTT_0384c		I		Phosphatidylserine decarboxylase proenzyme	3.06
FTN_RS02630	FTN_0506	FTL_0478	FTT_0408	*gcvH*	E	Glycine cleavage system	Glycine cleavage system protein H	0.34
FTN_RS02635	FTN_0507	FTL_0479	FTT_0409	*gcvP1*	E	Glycine cleavage system	Glycine dehydrogenase	0.44
FTN_RS02640	FTN_0508	FTL_0480	FTT_0410	*gcvP2*	E	Glycine cleavage system	Glycine dehydrogenase (decarboxylating)	0.48
FTN_RS02995	FTN_0579	FTL_1573	FTT_0488c		PRGE	MFS	MFS transporter	0.28
FTN_RS03110	FTN_0601	FTL_1546	FTT_0511	*pdxS*	H		Pyridoxal 5'-phosphate synthase lyase subunit	0.31
FTN_RS03115	FTN_0602	FTL_1545	FTT_0512	*pdxT*	H		Pyridoxal 5'-phosphate synthase glutaminase subunit	0.30
FTN_RS03205	FTN_0620	FTL_1528	FTT_0708		PRGE	MFS	MFS transporter	0.29
FTN_RS03265	FTN_0632	FTL_1503	FTT_0720c	*dgt*	F		HD domain-containing protein	0.47
FTN_RS03295	FTN_0637	FTL_1511	FTT_0726c	*ugpQ*	C		Glycerophosphoryl diester phosphodiesterase	0.32
FTN_RS03825	FTN_0741	FTL_1345	FTT_0567c		E	MFS	MFS transporter	0.34
FTN_RS04035	FTN_0783	NA	FTT_1091		Q		Cysteine hydrolase	0.18
FTN_RS04040	NA	FTL_1113	FTT_1090		H		Hypothetical protein	0.22
FTN_RS04045	FTN_0785	FTL_1114	FTT_1089		Q		Cysteine hydrolase	0.24
FTN_RS04170	FTN_0808	NA	FTT_0930c		Q		Acetoacetate decarboxylase	2.13
FTN_RS04250	FTN_0824	NA	FTT_0947c		PRGE	MFS	MFS transporter	2.17
FTN_RS04430	FTN_0860	FTL_1220	FTT_0979c		E		Amino acid permease	3.32
FTN_RS04435	FTN_0861	FTL_1219	FTT_0980		H		Hypothetical protein	0.30
FTN_RS04475	FTN_0869	FTL_1213	FTT_0989		E		DUF3857 domain-containing	0.31

							protein	
FTN_RS04605	FTN_0894	FTL_1074	FTT_1016c		E		Arylesterase	2.24
FTN_RS04685	FTN_0910	NA	FTT_1032		G	MFS	MFS transporter	0.26
FTN_RS04690	FTN_0911	FTL_1052	FTT_1033		G		Alpha-glucosidase	0.31
FTN_RS05140	FTN_0997	FTL_0963	FTT_0686c		E	MFS	MFS transporter	0.47
FTN_RS05315	FTN_1032	FTL_0925	FTT_0651		E		Peptide transporter	0.49
FTN_RS05535	FTN_1076	FTL_0875	FTT_0607	*ispG*	I		4-hydroxy-3-methylbut-2-en-1-yl diphosphate synthase	2.06
FTN_RS05695	FTN_1109	FTL_0834	FTT_1127		P		Rhodanese	0.49
FTN_RS05775	FTN_1125	NA	FTT_1144		RQI		KR domain-containing protein	0.46
FTN_RS05790	FTN_1128	FTL_0808	FTT_1147c		H		Bifunctional phosphopantothenoylcysteine decarboxylase/phosphopantothenate--cysteine ligase CoaBC	0.49
FTN_RS06315	FTN_1231	FTL_0732	FTT_1212c	*gloA*	E		Lactoylglutathione lyase	2.06
FTN_RS06620	FTN_1292	FTL_1183	FTT_1277c		RE		Sodium:solute symporter	2.69
FTN_RS06805	FTN_1329	FTL_1149	FTT_1365c	*fbaA*	G	Glycolytic and gluconeogenic pathway	Fructose-bisphosphate aldolase class II	0.29
FTN_RS06810	FTN_1330	FTL_1148	FTT_1366c	*pyk*	G	Glycolytic and gluconeogenic pathway	Pyruvate kinase	0.34
FTN_RS06815	FTN_1331	FTL_1147	FTT_1367c	*pgk*	G	Glycolytic and gluconeogenic pathway	Phosphoglycerate kinase	0.23
FTN_RS06820	FTN_1332	FTL_1146	FTT_1368c	*gapA*	G	Glycolytic and gluconeogenic pathway	Type I glyceraldehyde-3-phosphate dehydrogenase	0.18
FTN_RS06825	FTN_1333	FTL_1145	FTT_1369c	*tktA*	G	Glycolytic and gluconeogenic pathway	Transketolase	0.22

FTN_RS06960	FTN_1360	FTL_0665	FTT_1398c		E		FMN-binding glutamate synthase family protein	0.45
FTN_RS07170	FTN_1401	FTL_0627	FTT_1431		E		LysE family translocator	0.49
FTN_RS07215	FTN_1410	FTL_0617	FTT_1441	*bfr*	P		Bacterioferritin	0.49
FTN_RS07470	FTN_1460	FTL_0557	FTT_1552		I		Acyl-CoA desaturase	0.45
FTN_RS07540	FTN_1474	NA	FTT_1565c	*bglX*	G		Glycoside hydrolase family 3 protein	0.17
FTN_RS07795	FTN_1523	FTL_0280	FTT_1513		E	APC	APC family permease	0.47
FTN_RS08130	FTN_1589	FTL_1648	FTT_0126	*oppF*	E	ABC	ABC transporter ATP-binding protein	0.39
FTN_RS08135	FTN_1590	FTL_1648	FTT_0125	*oppD*	PE	ABC	ABC transporter ATP-binding protein	0.40
FTN_RS08140	FTN_1591	NA	FTT_0124	*oppC*	PE	ABC	ABC transporter permease	0.36
FTN_RS08145	FTN_1592	NA	FTT_0123	*oppB*	PE	ABC	Peptide ABC transporter	0.32
FTN_RS08150	FTN_1593	FTL_1653	FTT_0122	*oppA*	E	ABC	Peptide ABC transporter substrate-binding protein	0.43
FTN_RS08315	FTN_1620	FTL_1765	FTT_0091c	*appB*	C		Cytochrome d ubiquinol oxidase subunit II	0.49
FTN_RS08485	FTN_1651	FTL_1800	FTT_0059	*atpE*	C		F0F1 ATP synthase subunit C	0.48
FTN_RS08865	FTN_1727	NA	NA	*dapD*	E		2,3,4,5-tetrahydropyridine-2,6-dicarboxylate N-succinyltransferase	0.22
FTN_RS08875	FTN_1729	NA	NA	*dapB*	E		Dihydrodipicolinate reductase	0.21
FTN_RS08880	FTN_1730	NA	NA	*lysC*	E		Lysine-sensitive aspartokinase 3	0.31
FTN_RS08915	FTN_1737	NA	FTT_1775c	*clcA*	P	CLC	Voltage-gated ClC-type chloride channel	0.42
FTN_RS08975	FTN_1748	FTL_0089	FTT_1764c		C		Ferredoxin family protein	0.40

FTN_RS09055	FTN_1763	FTL_1948	FTT_1783	PRGE	MFS	MFS transporter	2.31
FTN_RS09320	FTN_0990	NA	NA	C		FAD-binding oxidoreductase	2.08
				**Poorly**	**characterized**		
FTN_RS00125	FTN_0025	NA	NA	S		Hypothetical protein	2.06
FTN_RS00220	FTN_0042	NA	NA	S		Hypothetical protein	5.29
FTN_RS00225	FTN_0043	NA	NA	S		Hypothetical protein	4.01
FTN_RS00230	FTN_0044	NA	NA	S		Hypothetical protein	2.64
FTN_RS00235	FTN_0045	NA	NA	S		Hypothetical protein	2.80
FTN_RS00240	FTN_0046	NA	NA	S		Hypothetical protein	2.40
FTN_RS00245	FTN_0047	NA	NA	S		Hypothetical protein	2.35
FTN_RS00250	FTN_0048	NA	NA	S		Hypothetical protein	2.09
FTN_RS00255	FTN_0049	NA	NA	S		Hypothetical protein	2.25
FTN_RS00335	FTN_0065	NA	NA	S		Hypothetical protein	0.33
FTN_RS00410	FTN_0080	FTL_0171	FTT_0240	R		16S rRNA (cytidine(1402)-2'-O)-methyltransferase	2.13
FTN_RS00530	FTN_0103	NA	FTT_1682	S		Hypothetical protein	0.26
FTN_RS00670	FTN_0131	FTL_0136	FTT_0254c	S		Hypothetical protein	0.24
FTN_RS00795	FTN_0155	FTL_1916	FTT_0179	R		DUF4131 domain-containing protein	2.14
FTN_RS01465	FTN_0284	NA	NA	R		Hypothetical protein	2.08
FTN_RS01755	FTN_0340	FTL_1639	FTT_0825c	S		Hypothetical protein	0.18
FTN_RS01980	FTN_0381	NA	FTT_0852	S		Hypothetical protein	0.27
FTN_RS02405	FTN_0463	FTL_0403	Na	S		Hypothetical protein	2.26
FTN_RS02420	FTN_0466	FTL_0442	FTT_1292c	S		Hypothetical protein	3.02
FTN_RS02590	NA	NA	NA	S		Hypothetical protein	0.49
FTN_RS02880	NA	FTL_1599	FTT_0465	S		Hypothetical protein	0.44

FTN_RS03030	FTN_0586	NA	FTT_0495	S	Hypothetical protein	2.24
FTN_RS03085	NA	FTL_1551	FTT_0506c	S	Hypothetical protein	2.72
FTN_RS03180	FTN_0615	FTL_1532	FTT_0704	S	Hypothetical protein	2.08
FTN_RS03325	FTN_0643	FTL_1494	FTT_1334c	S	Hypothetical protein	0.48
FTN_RS03380	FTN_0654	FTL_1485	FTT_1324	S	Hypothetical protein	0.40
FTN_RS03620	FTN_0701	FTL_1377	FTT_0736	R	Alpha/beta hydrolase	2.71
FTN_RS03715	FTN_0719	NA	FTT_0747c	S	Hypothetical protein	0.48
FTN_RS03820	FTN_0740	FTL_1347	FTT_0566	S	Hypothetical protein	0.42
FTN_RS03935	FTN_0763	NA	NA	R	Acyl-CoA thioesterase	2.03
FTN_RS03995	FTN_0775	FTL_1101	FTT_1099c	S	Hypothetical protein	2.19
FTN_RS04310	FTN_0836	FTL_1246	FTT_0957c	R	Kinase	2.69
FTN_RS04315	FTN_0837	NA	NA	S	Hypothetical protein	2.52
FTN_RS04450	FTN_0864	FTL_1217	NA	S	Hypothetical protein	0.30
FTN_RS04520	FTN_0878	FTL_1202	FTT_0998	S	Hypothetical protein	3.32
FTN_RS04835	FTN_0936	NA	NA	S	Hypothetical protein	2.47
FTN_RS05180	FTN_1005	FTL_0954	FTT_0678c	S	Hypothetical protein	2.73
FTN_RS05260	FTN_1021	NA	FTT_0662c	S	Hypothetical protein	0.17
FTN_RS05495	FTN_1068	NA	FTT_0613c	S	Hypothetical protein	0.30
FTN_RS05510	FTN_1071	FTL_0880	FTT_0612	S	Hypothetical protein	0.47
FTN_RS05565	FTN_1083	FTL_0860	FTT_0596c	S	Hypothetical protein	2.08
FTN_RS05655	FTN_1101	NA	NA	S	Hypothetical protein	0.26
FTN_RS05660	NA	NA	NA	S	Hypothetical protein	0.10
FTN_RS05665	FTN_1103	NA	NA	S	Hypothetical protein	0.05
FTN_RS05670	FTN_1104	NA	NA	S	Hypothetical protein	0.04
FTN_RS05760	FTN_1122	FTL_0816	FTT_1140	S	Hypothetical protein	0.25
FTN_RS05765	FTN_1123	FTL_0814	FTT_1141	S	Hypothetical protein	0.45

FTN_RS05770	FTN_1124	NA	FTT_1143	S	Hypothetical protein	0.37
FTN_RS05780	FTN_1126	FTL_0810	FTT_1145	R	Hypothetical protein	0.43
FTN_RS05820	FTN_1134	NA	FTT_1153c	S	Hypothetical protein	2.06
FTN_RS05865	NA	NA	NA	S	Hypothetical protein	0.25
FTN_RS05875	FTN_1144	NA	FTT_1163c	S	Hypothetical protein	2.10
FTN_RS06290	FTN_1227	NA	FTT_0783	R	MBL fold metallo-hydrolase	2.08
FTN_RS06300	FTN_1229	FTL_1443	FTT_0781c	R	TIGR00730 family Rossman fold protein	2.28
FTN_RS06305	NA	NA	NA	S	Hypothetical protein	2.32
FTN_RS06310	FTN_1230	NA	FTT_1211c	S	Hypothetical protein	2.47
FTN_RS06465	FTN_1261	NA	NA	S	Hypothetical protein	0.12
FTN_RS06830	FTN_1334	FTL_1144	FTT_1370	S	Hypothetical protein	0.38
FTN_RS07420	FTN_1450	FTL_0570	FTT_1541c	S	Hypothetical protein	0.37
FTN_RS07425	FTN_1451	FTL_0569	FTT_1542c	S	Hypothetical protein	0.20
FTN_RS07460	FTN_1458	FTL_0559	FTT_1550	S	Hypothetical protein	0.42
FTN_RS07465	FTN_1459	FTL_0558	FTT_1551	R	KR domain-containing protein	0.40
FTN_RS07530	FTN_1472	FTL_0544	FTT_1564	S	Hypothetical protein	3.06
FTN_RS08320	NA	FTL_1766	FTT_0090c	S	Hypothetical protein	0.46
FTN_RS08430	NA	NA	FTT_0069c	S	Hypothetical protein	0.38
FTN_RS08910	FTN_1736	NA	FTT_1776c	S	Hypothetical protein	2.72
FTN_RS08940	FTN_1741	FTL_0097	FTT_1771	S	Hypothetical protein	0.33
FTN_RS09065	FTN_1765	FTL_1950	FTT_1786	R	Hypothetical protein	3.86
FTN_RS09160	NA	NA	NA	S	Hypothetical protein	0.20
FTN_RS09165	NA	NA	NA	S	Hypothetical protein	0.23
FTN_RS09180	NA	NA	NA	S	Hypothetical protein	0.44
FTN_RS09185	NA	NA	NA	R	Membrane protein	0.48

FTN_RS09245	NA	NA	NA		S	Hypothetical protein	0.19
FTN_RS09325	NA	NA	NA		S	Hypothetical protein	0.44
FTN_RS09365	NA	NA	NA		S	Hypothetical protein	2.45
				**FPI**			
FTN_RS06705	FTN_1309	FTL_0126	FTT_1344	*pdpA*	FPI	Pathogenicity determinant protein A	0.32
FTN_RS06710	FTN_1310	FTL_0125	FTT_1345	*pdpB*	FPI	Pathogenicity determinant protein B	0.30
FTN_RS06715	FTN_1311	FTL_0124	FTT_1346	*iglE*	FPI	Intracellular growth locus E protein	0.30
FTN_RS06720	FTN_1312	FTL_0123	FTT_1347	*vgrG*	FPI	Valine-glycine repeat protein G	0.31
FTN_RS06730	FTN_1314	FTL_0121	FTT_1349	*iglG*	FPI	Intracellular growth locus G protein	0.41
FTN_RS06735	FTN_1315	FTL_0120	FTT_1350	*iglH*	FPI	Intracellular growth locus H protein	0.40
FTN_RS06740	FTN_1316	FTL_0119	FTT_1351	*dotU*	FPI	Defect in organelle trafficking	0.36
FTN_RS06745	FTN_1317	FTL_0118	FTT_1352	*iglL*	FPI	Intracellular growth locus L protein	0.32
FTN_RS06750	FTN_1318	FTL_0117	FTT_1353	*iglJ*	FPI	Intracellular growth locus J protein	0.34
FTN_RS06755	FTN_1319	FTL_0116	FTT_1354	*pdpC*	FPI	Pathogenicity determinant protein C	0.42
FTN_RS06765	FTN_1321	FTL_0114	FTT_1356c	*iglD*	FPI	Intracellular growth locus D protein	0.28
FTN_RS06770	FTN_1322	FTL_0113	FTT_1357c	*iglC*	FPI	Intracellular growth locus C protein	0.22
FTN_RS06775	FTN_1323	FTL_0112	FTT_1358c	*iglB*	FPI	Intracellular growth locus B protein	0.33
FTN_RS06780	FTN_1324	FTL_0111	FTT_1359c	*iglA*	FPI	Intracellular growth locus A protein	0.48
FTN_RS06785	FTN_1325	FTL_0110	FTT_1360c	*pdpD*	FPI	Pathogenicity determinant protein D	0.33

[1]COG category: **J**, Translation, ribosomal structure and biogenesis; **K**, Transcription; **L**, Replication, recombination and repair; **D**, Cell cycle control, cell division, chromosome partitioning; **V**, Defense mechanisms; **T**, Signal transduction mechanisms; **M**, Cell wall/membrane/envelope biogenesis; **W**, Extracellular structures; **U**, Intracellular trafficking, secretion, and vesicular transport; **O**, Posttranslational modification, protein turnover, chaperones; **C**, Energy production and conversion; **G**, Carbohydrate transport and metabolism; **E**, Amino acid transport and metabolism; **F**, Nucleotide transport and metabolism; **I**, Lipid transport and metabolism; **P**, Inorganic ion transport and metabolism; **Q**, Secondary metabolites biosynthesis, transport and catabolism; **R**, General function prediction only; **S**, Function unknown; **FPI**, *Francisella* pathogenicity island. [2]Transport family names: **ABC**, ATP-binding cassette superfamily; **APC**, amino acid-polyamine-organocation family; **MFS**, major facilitator

superfamily; **CLC**, Chloride channel family.
