# Supplementary material for: Transcription Elongation Factor GreA Plays a Key Role in Cellular Invasion and Virulence of Francisella tularensis subsp. novicida
Source: Sci Rep. 2018 May 2;8:6895. doi: 10.1038/s41598-018-25271-5 (PMC5932009; doi:10.1038/s41598-018-25271-5)
Supplement: Supplementary file 6 — Table S3 [file 41598_2018_25271_MOESM6_ESM.pdf]

Transcription Elongation Factor GreA Plays a Key Role in Cellular Invasion and Virulence of *Francisella tularensis* subsp. *novicida*

Guolin Cui[1], Jun Wang[1], Xinyi Qi[1], Jingliang Su[1*]

Table S3 The differentially expressed genes previously shown to be involved in cellular invasion and/or intracellular growth and/or virulence in the Δ*greA* mutant compared with the wild-type U112 in this study

Locus tag	Gene name[1]	Gene product	Fold change by RNAseq	COG category	Phenotypic defects	References
			**FPI**			
FTN_1309	*pdpA*	Pathogenicity determinant protein A	0.32	FPI	Cellular invasion defect and attenuation in mice	1-3
FTN_1310	*pdpB*	Pathogenicity determinant protein B	0.30	FPI	Intracellular growth defect and attenuation in mice	1,2,4-6
FTN_1311	*iglE*	Intracellular growth locus E protein	0.30	FPI	Intracellular growth defect and attenuation in mice	1,2,6
FTN_1312	*vgrG*	Valine-glycine repeat protein G	0.31	FPI	Intracellular growth defect and attenuation in mice	1,2,6,7
FTN_1314	*iglG*	Intracellular growth locus G protein	0.41	FPI	Intracellular growth defect and attenuation in mice	1,2,6,8,9
FTN_1315	*iglH*	Intracellular growth locus H protein	0.40	FPI	Intracellular growth defect and attenuation in mice	1,2,6,8,9
FTN_1316	*dotU*	Defect in organelle trafficking	0.36	FPI	Intracellular growth defect and attenuation in	1,2,6

					mice	
FTN_1317	*iglI*	Intracellular growth locus L protein	0.32	FPI	Intracellular growth defect and attenuation in mice	1,2,6
FTN_1318	*iglJ*	Intracellular growth locus J protein	0.34	FPI	Intracellular growth defect and attenuation in mice	1,2,6
FTN_1319	*pdpC*	Pathogenicity determinant protein C	0.42	FPI	Attenuation in mice and *D. melanogaster*	1,4,9
FTN_1321	*iglD*	Intracellular growth locus D protein	0.28	FPI	Intracellular growth defect, attenuation in mice and *D. melanogaster*	1,2,4,6-10
FTN_1322	*iglC*	Intracellular growth locus C protein	0.22	FPI	Cellular invasion and intracellular growth defect, attenuation in mice and *D. melanogaster*	1-4,6,8,9,11
FTN_1323	*iglB*	Intracellular growth locus B protein	0.33	FPI	Intracellular growth defect and attenuation in mice	1,2,6,10,11
FTN_1324	*iglA*	Intracellular growth locus A protein	0.48	FPI	Intracellular growth defect and attenuation in mice	1,2,6,11
FTN_1325	*pdpD*	Pathogenicity determinant protein D	0.33	FPI	Intracellular growth defect and attenuation in mice	1,8,9
			**Metabolism**			
FTN_0008		EamA/RhaT family transporter	0.45	RGE	Intracellular growth defect and attenuation in mice	7-9
FTN_0078	*aroE*	Shikimate dehydrogenase	2.19	E	Intracellular growth defect	4
FTN_0217	*lldD*	Alpha-hydroxy-acid oxidizing protein	2.43	C	Intracellular growth defect, attenuation in mice and *D. melanogaster*	4,7-9
FTN_0405	*sodC*	Superoxide dismutase	0.30	P	Intracellular growth defect and attenuation in mice	12
FTN_0406		Sterol desaturase family protein	0.46	I	Intracellular growth defect	8,9

FTN_0506	*gcvH*	Glycine cleavage system protein H	0.34	E	Attenuation in mice	[1]
FTN_0507	*gcvP1*	Glycine cleavage system protein P, subunit 1	0.44	E	Intracellular growth defect, attenuation in mice and *D. melanogaster*	[1,8,9]
FTN_0579		MFS transporter	0.28	PRGE	Intracellular growth defect and attenuation in *D. melanogaster*	[8,9]
FTN_0620		MFS transporter	0.29	PRGE	Intracellular growth defect and attenuation in mice	[8,9,11]
FTN_0741		MFS transporter	0.34	E	Intracellular growth defect and attenuation in *D. melanogaster*	[8,9]
FTN_0783		Cysteine hydrolase	0.18	Q	Intracellular growth defect	[4]
FTN_0824		MFS transporter	2.17	PRGE	Intracellular growth defect and attenuation in mice	[4,7-9]
FTN_0861		Hypothetical protein	0.30	H	Intracellular growth defect	[4,8,9]
FTN_0910		MFS transporter	0.26	G	Intracellular growth defect	[8,9]
FTN_0997		MFS transporter	0.47	E	Intracellular growth defect and attenuation in *D. melanogaster*	[8,9]
FTN_1231	*gloA*	Lactoylglutathione lyase	2.06	E	Intracellular growth defect	[8,9]
FTN_1292		Sodium:solute symporter	2.69	RE	Attenuation in mice	[7]
FTN_1329	*fbaA*	Fructose-bisphosphate aldolase	0.29	G	Intracellular growth defect	[8,9,13]
FTN_1333	*tktA*	Transketolase	0.22	G	Intracellular growth defect, attenuation in mice and *D. melanogaster*	[5,8,9]
FTN_1410	*bfr*	Bacterioferritin	0.49	P	Attenuation in mice	[11]
FTN_1523		APC family permease	0.47	E	Intracellular growth defect	[8,9]
FTN_1593	*oppA*	Peptide ABC transporter substrate-binding protein	0.43	E	Intracellular growth defect, attenuation in mice and *D. melanogaster*	[8,9]
FTN_1620	*appB*	Cytochrome d ubiquinol oxidase subunit	0.49	C	Intracellular growth defect, attenuation in mice	[8,9]

		II			and *D. melanogaster*	
FTN_1729	*dapB*	Dihydrodipicolinate reductase	0.21	E	Intracellular growth defect	[8,9]
FTN_1730	*lysC*	Lysine-sensitive aspartokinase 3	0.31	E	Intracellular growth defect	[8,9]
FTN_1763		MFS transporter	2.31	PRGE	Intracellular growth defect	[4]
			**Cellular processes and signaling**			
FTN_0289	*proQ*	Activator of osmoprotectant transporter ProP	2.54	T	Attenuation in mice	[1]
FTN_0431		Type IV pili glycosylation protein	0.46	M	Attenuation in mice	[1]
FTN_0767	*betT*	BCCT family transporter	0.48	M	Intracellular growth defect	[8,9]
FTN_1064	*phoH*	PhoH family protein	0.48	T	Intracellular growth defect, attenuation in mice and *D. melanogaster*	[1,8,9]
FTN_1186	*pepO*	M13 family peptidase	0.16	O	Intracellular growth defect and attenuation in *D. melanogaster*	[4,8,9]
FTN_1220		Sugar transferase	2.06	M	Intracellular growth defect and attenuation in mice	[1,4]
FTN_1252		Linear amide C-N hydrolase	2.14	M	Intracellular growth defect	[14]
FTN_1551	*ampD*	1,6-anhydro-N-acetylmuramyl-L-alanine amidase AmpD	0.33	V	Attenuation in mice	[1]
FTN_1756		Thioredoxin-dependent thiol peroxidase	2.05	O	Attenuation in mice	[7]
			**Information storage and processing**			
FTN_0010		Terminase small subunit	4.56	L	Intracellular growth defect	[8,9]
FTN_0465		Threonylcarbamoyl-AMP synthase	4.58	J	Intracellular growth defect and attenuation in *D. melanogaster*	[8,9]
FTN_0603	*mutM*	Formamidopyrimidine-DNA glycosylase	2.06	L	Intracellular growth defect	[4]
FTN_0838	*xthA*	Exodeoxyribonuclease III	2.07	L	Intracellular growth defect	[4,8,9]

FTN_1063	*miaB*	tRNA (N6-isopentenyl adenosine(37)-C2)-methylthiotransferase	0.43	J	Intracellular growth defect and attenuation in mice	7,15
FTN_1612		Transposase	2.14	L	Intracellular growth defect and attenuation in *D. melanogaster*	8,9
			**Poorly characterized**			
FTN_0043		Type VI secretion system contractile sheath large subunit	4.01	S	Intracellular growth defect	8,9
FTN_0044		Hypothetical protein	2.64	S	Intracellular growth defect	8,9
FTN_0045		Hypothetical protein	2.80	S	Attenuation in mice	7
FTN_0048		Hypothetical protein	2.09	S	Intracellular growth defect and attenuation in *D. melanogaster*	9
FTN_0080		16S rRNA (cytidine(1402)-2'-O)-methyltransferase	2.13	S	Intracellular growth defect	8,9
FTN_0155		DUF4131 domain-containing protein	2.14	R	Intracellular growth defect	8,9
FTN_0340		Hypothetical protein	0.18	S	Intracellular growth defect and attenuation in mice	7-9
FTN_0466		YigZ family protein	3.02	S	Intracellular growth defect and attenuation in *D. melanogaster*	8,9
FTN_0701		Alpha/beta hydrolase	2.71	R	Intracellular growth defect	8,9
FTN_0740		Hypothetical protein	0.42	S	Attenuation in mice	7
FTN_0836		Kinase	2.69	R	Intracellular growth defect	8,9
FTN_0878		Hypothetical protein	3.32	S	Intracellular growth defect	8,9
FTN_1071		Hypothetical protein	0.47	S	Intracellular growth defect	8,9
FTN_1103		Hypothetical protein	0.05	S	Intracellular growth defect	8,9
FTN_1123		Photosystem reaction center subunit H	0.45	S	Intracellular growth defect and attenuation in *D. melanogaster*	8,9

FTN_1261	Hypothetical protein	0.12	S	Intracellular growth defect and attenuation in *D. melanogaster*	8,9
FTN_1334	Hypothetical protein	0.38	S	Intracellular growth defect	8,9
FTN_1459	KR domain-containing protein	0.40	R	Intracellular growth defect and attenuation in mice	7-9
FTN_1472	Polyphosphate kinase 2	3.06	S	Intracellular growth defect	15
FTN_1736	DUF3568 domain-containing protein	2.72	S	Intracellular growth defect	8,9
FTN_1765	Hypothetical protein	3.86	R	Intracellular growth defect	8,9

[1]The Francisella pathogenicity island (FPI) nomenclature proposed by Ludu[16] and Barker[17] was used.

References

1 Weiss, D. S. *et al.* In vivo negative selection screen identifies genes required for Francisella virulence. *Proc Natl Acad Sci U S A* **104**, 6037-6042, doi:10.1073/pnas.0609675104 (2007).

2 Ahlund, M. K., Ryden, P., Sjostedt, A. & Stoven, S. Directed screen of Francisella novicida virulence determinants using Drosophila melanogaster. *Infect. Immun.* **78**, 3118-3128, doi:10.1128/IAI.00146-10 (2010).

3 Law, H. T. *et al.* IglC and PdpA Are Important for Promoting Francisella Invasion and Intracellular Growth in Epithelial Cells. *PLoS One* **9**, e104881 (2014).

4 Moule, M. G., Monack, D. M. & Schneider, D. S. Reciprocal Analysis of Francisella novicida Infections of a Drosophila melanogaster Model Reveal Host-Pathogen Conflicts Mediated by Reactive Oxygen and imd-Regulated Innate Immune Response. *PLoS Pathog.* **6**, e1001065 (2010).

5 Tempel, R., Lai, X. H., Crosa, L., Kozlowicz, B. & Heffron, F. Attenuated Francisella novicida transposon mutants protect mice against wild-type challenge. *Infect. Immun.* **74**, 5095-5105, doi:10.1128/IAI.00598-06 (2006).

6 Llewellyn, A. C., Jones, C. L., Napier, B. A., Bina, J. E. & Weiss, D. S. Macrophage replication screen identifies a novel Francisella hydroperoxide resistance protein involved in virulence. *PLoS One* **6**, e24201, doi:10.1371/journal.pone.0024201 (2011).

7 PS, K. *et al.* Genome-wide screen in Francisella novicida for genes required for pulmonary and systemic infection in mice. *Infect. Immun.* **77**, 232 (2009).

8 Asare, R. Molecular Complexity Orchestrates Modulation of Phagosome Biogenesis and Escape to the Cytosol of macrophages by Francisella tularensis. *Environ Microbiol* **12**, 2559–2586 (2010).

9 Asare, R., Akimana, C., Jones, S. & Abu Kwaik, Y. Molecular bases of proliferation of Francisella tularensis in arthropod vectors. *Environ Microbiol* **12**, 2587-

2612, doi:10.1111/j.1462-2920.2010.02230.x (2010).

10 Kadzhaev, K. *et al.* Identification of genes contributing to the virulence of Francisella tularensis SCHU S4 in a mouse intradermal infection model. *PLoS One* **4**, e5463, doi:10.1371/journal.pone.0005463 (2009).

11 Su, J. *et al.* Genome-wide identification of Francisella tularensis virulence determinants. *Infect. Immun.* **75**, 3089-3101, doi:10.1128/IAI.01865-06 (2007).

12 Melillo, A. A. *et al.* Identification of Francisella tularensis live vaccine strain CuZn superoxide dismutase as critical for resistance to extracellularly generated reactive oxygen species. *J Bacteriol* **191**, 6447-6456, doi:10.1128/jb.00534-09 (2009).

13 Ziveri, J. *et al.* The metabolic enzyme fructose-1,6-bisphosphate aldolase acts as a transcriptional regulator in pathogenic Francisella. *Nat Commun* **8**, 853, doi:10.1038/s41467-017-00889-7 (2017).

14 Qin, A. & Mann, B. J. Identification of transposon insertion mutants of Francisella tularensis tularensis strain Schu S4 deficient in intracellular replication in the hepatic cell line HepG2. *BMC Microbiol.* **6**, 69, doi:10.1186/1471-2180-6-69 (2006).

15 Maier, T. M. *et al.* Identification of Francisella tularensis Himar1-based transposon mutants defective for replication in macrophages. *Infect. Immun.* **75**, 5376-5389, doi:10.1128/IAI.00238-07 (2007).

16 Ludu, J. S. *et al.* The Francisella Pathogenicity Island Protein PdpD Is Required for Full Virulence and Associates with Homologues of the Type VI Secretion System. *J Bacteriol* **190**, 4584 (2008).

17 Barker, J. R. & Chong, A. T. The Francisella tularensis pathogenicity island encodes a secretion system that is required for phagosome escape and virulence. *Mol Microbiol* **74**, 1459-1470 (2009).
