# Supplementary material for: Transcription Elongation Factor GreA Plays a Key Role in Cellular Invasion and Virulence of Francisella tularensis subsp. novicida
Source: Sci Rep. 2018 May 2;8:6895. doi: 10.1038/s41598-018-25271-5 (PMC5932009; doi:10.1038/s41598-018-25271-5)
Supplement: Supplementary file 7 — Table S4 [file 41598_2018_25271_MOESM7_ESM.pdf]

# Transcription Elongation Factor GreA Plays a Key Role in Cellular Invasion and Virulence of *Francisella tularensis* subsp. *novicida*

Guolin Cui[1], Jun Wang[1], Xinyi Qi[1], Jingliang Su[1*]

**Table S4 The differentially expressed genes previously shown to be involved in cellular invasion in Δ*greA* mutant compared with the wild-type U112 in this study**

| Locus tag | Gene name | Species | Cell line | Fold change by RNAseq | Reference |
|---|---|---|---|---|---|
| FTN_1104 | | *Francisella tularensis* | Murine BNL CL.2 hepatocyte | 0.04 | 1 |
| FTN_1186 | *pepO* | *Porphyromonas gingivalis* | Human HeLa cell | 0.16 | 2 |
| FTN_1309 | *pdpA* | *Francisella tularensis* | Murine BNL CL.2 hepatocyte | 0.32 | 3 |
| FTN_1322 | *iglC* | *Francisella tularensis* | Murine BNL CL.2 hepatocyte | 0.22 | 3 |
| FTN_1551 | *ampD* | *Salmonella enterica* | Murine J774 macrophage | 0.33 | 4 |

## References


1. Lo, Y. S. Identification and characterization of Francisella tularensis proteins required for invasion and escape into non-phagocytic epithelial cells. [dissertationn/docter's thesis]. [Burnaby (British Columbia)]: Simon Fraser Univesity. (2017).
2. Ansai, T., Yu, W., Urnowey, S., Barik, S. & Takehara, T. Construction of a pepO gene-deficient mutant of Porphyromonas gingivalis : potential role of endopeptidase O in the invasion of host cells. *Mol Oral Microbiol* **18**, 398–400 (2003).
3. Law, H. T. *et al.* IglC and PdpA Are Important for Promoting Francisella Invasion and Intracellular Growth in Epithelial Cells. *PLoS One* **9**, e104881 (2014).
4. Folkesson, A., Eriksson, S., Andersson, M., Park, J. T. & Normark, S. Components of the peptidoglycan-recycling pathway modulate invasion and intracellular survival of Salmonella enterica serovar Typhimurium. *Cell Microbiol* **7**, 147-155, doi:10.1111/j.1462-5822.2004.00443.x (2005).
